# Pharmacological inhibition of LSD1 triggers myeloid differentiation by targeting GSE1 oncogenic functions in AML

**DOI:** 10.1038/s41388-021-02123-7

**Published:** 2021-12-03

**Authors:** Luciano Nicosia, Francesca Ludovica Boffo, Elena Ceccacci, Fabio Conforti, Isabella Pallavicini, Fabio Bedin, Roberto Ravasio, Enrico Massignani, Tim C. P. Somervaille, Saverio Minucci, Tiziana Bonaldi

**Affiliations:** 1 Department of Experimental Oncology, IEO, European Institute of Oncology IRCCS, Milan, 20139 Italy; 2 Leukaemia Biology Laboratory, Cancer Research UK Manchester Institute, The University of Manchester, Oglesby Cancer Research Centre Building, Manchester, M20 4GJ UK; 3 Department of Biosciences, University of Milan, Milan, 20133 Italy; 4 Department of Oncology and Haemato-Oncology, University of Milan, Milan, 20133 Italy

**Keywords:** Transcription, Acute myeloid leukaemia

## Abstract

The histone demethylase LSD1 is over-expressed in hematological tumors and has emerged as a promising target for anticancer treatment, so that several LSD1 inhibitors are under development and testing, in preclinical and clinical settings. However, the complete understanding of their complex mechanism of action is still unreached. Here, we unraveled a novel mode of action of the LSD1 inhibitors MC2580 and DDP-38003, showing that they can induce differentiation of AML cells through the downregulation of the chromatin protein GSE1. Analysis of the phenotypic effects of *GSE1* depletion in NB4 cells showed a strong decrease of cell viability in vitro and of tumor growth in vivo. Mechanistically, we found that a set of genes associated with immune response and cytokine-signaling pathways are upregulated by LSD1 inhibitors through GSE1-protein reduction and that LSD1 and GSE1 colocalize at promoters of a subset of these genes at the basal state, enforcing their transcriptional silencing. Moreover, we show that LSD1 inhibitors lead to the reduced binding of GSE1 to these promoters, activating transcriptional programs that trigger myeloid differentiation. Our study offers new insights into GSE1 as a novel therapeutic target for AML.

## Introduction

By catalyzing the removal of methyl groups from mono- and dimethylated forms of lysine 4 and lysine 9 of histone H3 (H3K4me1/me2 and H3K9me1/me2), the epigenetic enzyme lysine-specific histone demethylase 1 A (LSD1/KDM1A) has emerged as a major player in gene expression modulation in eukaryotes [1, 2]. In different cell types, this enzyme can act as either a transcriptional corepressor or coactivator, depending on the distinct set of interactions established [3]. Most frequently, LSD1 is embedded in transcriptional-repressive complexes such as CoREST and NuRD [4–6], where different subunits modulate LSD1 activity: CoREST confers to LSD1 the ability to bind nucleosomes, directs its demethylase activity toward H3K4me1/me2, and protects it from proteasomal degradation; HDACs create a hypoacetylated chromatin environment that stimulates LSD1 catalytic activity [6–8]. Less often, such as in the context of androgen (AR)- and estrogen (ER)-receptor-dependent transcription, LSD1 acts as a transcriptional coactivator by demethylating H3K9me1/me2 and thus promoting the downstream expression of AR- and ER- target genes [2, 9]. Some proteins, such as the protein-kinase C beta I (PKCbeta I) and PELP1, help directing the LSD1 demethylase activity toward H3K9 more than H3K4 [10, 11]. LSD1 also exhibits scaffolding activity, which facilitates recruitment of the repressive activity of histone deacetylase to sites on chromatin where SNAG-domain transcription factors such as GFI1 and GFI1B are bound [12, 13].

LSD1 is overexpressed in various solid and hematological tumors, where its increased levels are linked to poor prognosis [14–17]. Various studies demonstrated LSD1 contribution to the onset and progression of acute myeloid leukemia (AML), indicating that this enzyme can be a therapeutic target for the treatment of different AML subtypes [12, 18–20]. In particular, LSD1 has been shown to stimulate the clonogenic activity of leukemic stem cells (LSCs), trigger their oncogenic transcriptional programs [18], and also inhibit myeloid differentiation, as confirmed by the fact that LSD1 inhibition induces the activation of myeloid-lineage markers, such as CD11b and CD86 [12, 21]. All this evidence has prompted the drug-discovery field to develop LSD1 inhibitors as epigenetic anticancer drugs [22–25].

Because of the structural similarities of LSD1 with the monoamine oxidases (MAOs) MAO-A and MAO-B, inhibitors already known to target MAOs were chosen as starting scaffolds for the development of small molecules more specific and selective toward LSD1. In particular, the nonselective MAO inhibitor tranylcypromine (TCP)—which was the first compound described to efficiently inhibit LSD1 catalytic activity [26]—was the starting point for the design of MC2580 [27] and DDP-38003 [28], two probes with higher potency and selectivity toward LSD1 than LSD2, MAO-A, and MAO-B in in vitro assays. Moreover, these drugs were shown to inhibit tumor growth and induce differentiation when tested in murine AML blasts [27, 28].

Various studies have helped to dissect out the mechanisms of action (MoA) of LSD1 inhibitors in AML and solid tumors [29]. Recently, a number of publications have surprisingly shown that they can trigger AML differentiation not through the expected inhibition of its catalytic activity, but by altering LSD1 binding to some of its interactors. In particular, LSD1’s interaction with the transcription factors GFI1 [12, 13] and GFI1B [30] was found to be strongly affected by these drugs, leading to reduced cell proliferation and induction of myeloid differentiation in AML. These findings are particularly interesting because they highlighted for the first time the role of LSD1 for the assembly of multiprotein complexes on chromatin and suggested that small molecules originally developed to target LSD1 catalytic activity can physically inhibit this scaffolding function, with therapeutic implications.

In this study, we further elaborated on our recent results on the dynamic LSD1 interactome upon its pharmacological inhibition [13] and focused on GSE1, whose binding to LSD1 is reduced upon cell treatment with MC2580 and DDP-38003 inhibitors, as a consequence of its diminished protein expression. Few studies have investigated the molecular and cellular function of GSE1 in cancer so far. *GSE1* has been described as an oncogene overexpressed in solid tumors, such as breast, prostate, and gastric cancers, and its increased level has been linked with enhanced cell proliferation, colony formation, cell migration, invasion, and chemoresistance [31–34]. Recently, a tumor suppressor role has been ascribed to *GSE1* in neuroepithelial stem (NES) cells [35]. The activity of GSE1 in hematological malignancies, and AML in particular, has not been investigated, especially in the context of its physical and functional interaction with LSD1 [36–39].

Through molecular and phenotypic characterization of the effect of these drugs on GSE1 expression and its activity on chromatin, we demonstrate the oncogenic functions of GSE1 in AML and show that its drug-induced reduced expression enforces myeloid differentiation in AML, with relevant therapeutic implications.

## Results

### LSD1 inhibitors reduce the protein expression of GSE1 in AML

**Fig. 1:**
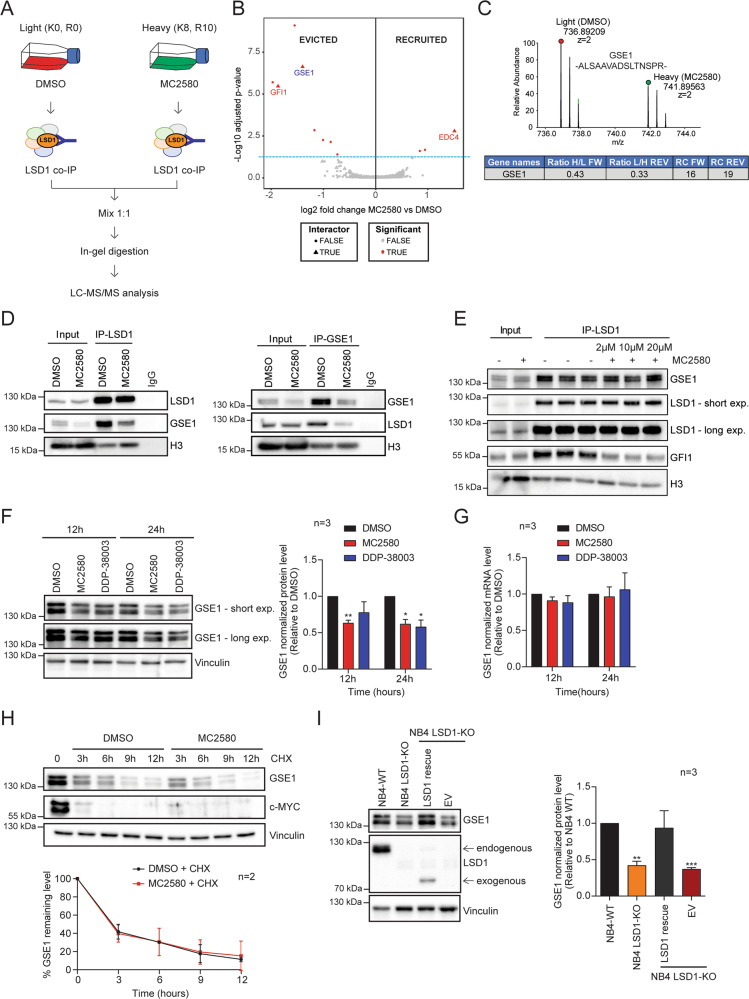
LSD1 inhibitors alter LSD1–GSE1 interaction by reducing GSE1 protein levels in AML. **A** Experimental design of the SILAC/LSD1 co-IP strategy setup to study the changes in LSD1 interactome upon 24 h treatment with 2 μΜ of MC2580, as described in ref. [13]. **B** Volcano plot displaying the alteration in the LSD1 interactome upon MC2580 treatment. Significantly evicted and recruited proteins are marked as red dots, while the proteins previously defined as specific LSD1 binders [13] are indicated by red triangles. The light-blue dashed line indicates the *p*-value threshold used to define the interactors modulated by the LSD1 inhibitors (*p* value < 0.05 calculated with limma [72]). Analysis of the evicted and recruited LSD1 interactors after pharmacological treatment is performed using the Differential Enrichment analysis of Proteomics data (DEP) R software package [40]. **C** *Upper panel:* MS1 spectrum of the SILAC peak doublet for the peptide 897-ALSAAVADSLTNSPR- 911 of GSE1 protein in the SILAC-LSD1 co-immuno-precipitated (co-IP) sample from NB4 cells treated for 24 h with 2 μΜ of MC2580 (heavy peak) and DMSO-treated control cells (light peak). *Lower panel:* Table displaying the H/L (forward replicate) and L/H (reverse replicate) ratios for GSE1 in SILAC-LSD1 co-IPs of NB4 cells treated with the compound MC2580 or DMSO. The table also shows the ratio count (RC) of each replicate, which corresponds to the number of peptides used for SILAC-based protein quantitation. The inhibitor was added to the heavy channel in the forward replicate and the light channel in the reverse one. **D** Western blot analysis of GSE1, LSD1, and H3 in LSD1 co-IPs (*left panel*) and GSE1 co-IPs (*right panel*), both in control and MC2580-treated cells (2 μΜ) for 24 h. Rabbit IgG was used as negative control co-IP. **E** Western blot analysis of GSE1, LSD1, GFI1, and H3 in in vitro LSD1 co-IPs using as input NB4 nuclear cell extract co-incubated for 6 h with increasing doses of MC2580 (2, 10 and 20 μM) and DMSO as control. **F** *Left panel:* Western blot analysis of GSE1 in NB4 cells treated with MC2580 (2 µM) DDP-38003 (2 µM) and control DMSO for 12 and 24 h. Vinculin was used as loading control. *Right panel:* Bar graph displaying the quantitation results of GSE1 protein, normalized over the Vinculin, in three independent replicates of NB4 cells treated with MC2580 (2 µM), DDP-38003 (2 µM), and DMSO. The results are plotted as fold change (FC) of GSE1 protein level in the treated samples over control (DMSO). The chart represents mean ± standard deviation (SD) (*n* = 3 biological replicates; one-sample T-test, **p value < 0.01, *p-value < 0.05). **G** RT-qPCR analysis on *GSE1* transcript in NB4 cells treated for 12 and 24 h with MC2580 (2 µM), DDP-38003 (2 µM), and DMSO as control. *GSE1* ct values are normalized against *GAPDH*. The results are plotted as FC of *GSE1* mRNA levels in LSD1 inhibitors-treated conditions over DMSO. The chart represents mean ± SD of three (*n* = 3) biological replicates. Primer list in Table S7. **H** *Upper panel:* Representative Western blot analysis of GSE1 and c-MYC in NB4 cells treated with cycloheximide (0.1 mg/ml) in combination with MC2580 (2 µM) and DMSO as control for 3, 6, 9, and 12 h. Vinculin was used as loading control. *Lower panel:* Line-plot displaying the percentage of GSE1 protein level normalized over Vinculin in cycloheximide-treated NB4 cells, together with 2 µM of MC2580 or DMSO as control. The chart represents mean ± SD (*n* = 2 biological replicates). **I** *Left panel:* Western blot analysis of GSE1 and LSD1 in NB4 WT, NB4 LSD1-KO, and LSD1-KO cells transduced with either a retroviral empty PINCO vector (EV) or a PINCO vector containing an exogenous, N-terminal truncated (172–833) form of LSD1 [13]. Vinculin was used as loading control. Arrows indicate the endogenous and the exogenous LSD1. *Right panel:* Bar graph displaying quantitation of GSE1 protein level, normalized over Vinculin level in three replicates of NB4 WT, NB4 LSD1-KO, and LSD1-KO cells, transduced with either an empty PINCO vector (EV) or a PINCO vector containing an exogenous, N-terminal truncated (172–833) form of LSD1 [13]. In all samples, the data are plotted as FC of GSE1 protein level compared with the NB4 WT, with mean ± SD from *n* = 3 biological replicates (one-sample T-test, **p value < 0.01, ***p-value < 0.001).

Using the Differential Enrichment analysis of Proteomics data (DEP) R software package [40], we reinterrogated the recently published dynamic LSD1 interactome upon treatment of NB4 acute promyelocytic leukemia (APL) cells with the LSD1 inhibitor MC2580 (Fig. 1A) [13] and we found that, in addition to the already-described GFI1, interaction with GSE1 protein was also significantly downregulated after drug treatment (Fig. 1B and C). We confirmed the MS results by western blot (WB) analysis, profiling GSE1 levels in the LSD1 co-IP and LSD1 in the reciprocal GSE1 co-IP, in control and MC2580-treated NB4 cells. While validating the reduced interaction between LSD1 and GSE1, inspection of both experiments led to the observation that GSE1 protein was decreased in the nuclear input of both co-IPs after LSD1 inhibition (Fig. 1D). Hence, in contrast to GFI1, we hypothesized that the reduced presence of GSE1 in the LSD1 co-IP was not due to the physical interference of a GSE1–LSD1 interaction by the drug, but rather due to diminished GSE1 expression.

We validated this hypothesis by performing an in vitro interaction assay in which NB4 nuclear extract was incubated with increasing concentrations of MC2580 or DMSO as control for 6 h, prior to carrying out the LSD1 co-IP. In line with our model, GSE1 was co-immunoprecipitated with LSD1 with the same efficiency in control- and MC2580-treated cells, while GFI1 was evicted by LSD1 after pharmacological inhibition, as previously reported [12, 13] (Fig. 1E).

We next assessed whether the reduction of GSE1 upon LSD1 inhibition occurred only at the protein or also at the transcript level. We treated NB4 cells with MC2580 and DDP-38003 for 12 h and 24 h and measured both GSE1 mRNA and protein by real-time quantitative PCR (RT-qPCR) and WB analysis, respectively. While the reduction of GSE1 protein was confirmed (Fig. 1F), *GSE1* transcript levels did not change significantly (Fig. 1G) ruling out transcriptional regulation of *GSE1* upon LSD1 inhibition. This finding was corroborated by the observation that an exogenous V5-tagged form of GSE1—cloned in two different lentiviral vectors and transduced in NB4 cells—also displayed a reduction 24 h post treatment with MC2580. Since the expression of exogenous *GSE1* was under the control of different promoters compared to that of the endogenous gene, this further excludes the possibility of transcriptional repression of *GSE1* by LSD1 inhibitors (Fig. S1). By assessing GSE1 levels also in other non-APL AML cell lines, including THP-1, SKNO-1, and OCI-AML2, as well as in an *MLL-*translocated primary patient sample (BB104) [12] (Fig. S2), we confirmed that the protein downregulation of GSE1 upon LSD1 inhibition was consistent and independent of cellular cytogenetic features.

In light of recent experimental evidence supporting a role of LSD1 in modulating the stability of target proteins independently from its catalytic activity [41–44], we next asked whether GSE1 reduction was the consequence of post translational mechanisms associated with protein destabilization. First, we assessed GSE1 protein levels at different time points upon treatment with cycloheximide (CHX), a translational elongation inhibitor. We observed a strong reduction 12 h post treatment, while c-MYC was efficiently degraded 3 h after CHX treatment, as already described [45] (Fig. S3). Next, we profiled GSE1 protein level at 3, 6, 9, and 12 h following CHX treatment in the presence or absence of MC2580 and observed that GSE1 protein stability was not altered when LSD1 was pharmacologically inhibited, suggesting that the diminished level of GSE1 after LSD1 inhibition is not due to the decreased stability or enhanced degradation of the protein, but likely to an inhibition of the translation process (Figs. 1H and S4).

Pharmacological data were corroborated by the analysis of LSD1 knockout (KO) cells, which showed diminished GSE1 protein compared with NB4 wild-type (WT) cells (Fig. 1I). Last, when we transduced LSD1 KO cells with a vector reexpressing an exogenous WT form of LSD1 [13], we observed that GSE1 protein was reestablished to a level significantly higher than in the cells transduced with an empty vector (EV) (Fig. 1I). This piece of data specifically links GSE1 protein reduction to the inhibition or depletion of LSD1, excluding off-target effects of the drugs.

### *GSE1* silencing reduces viability and in vivo tumor growth of NB4 cells

**Fig. 2:**
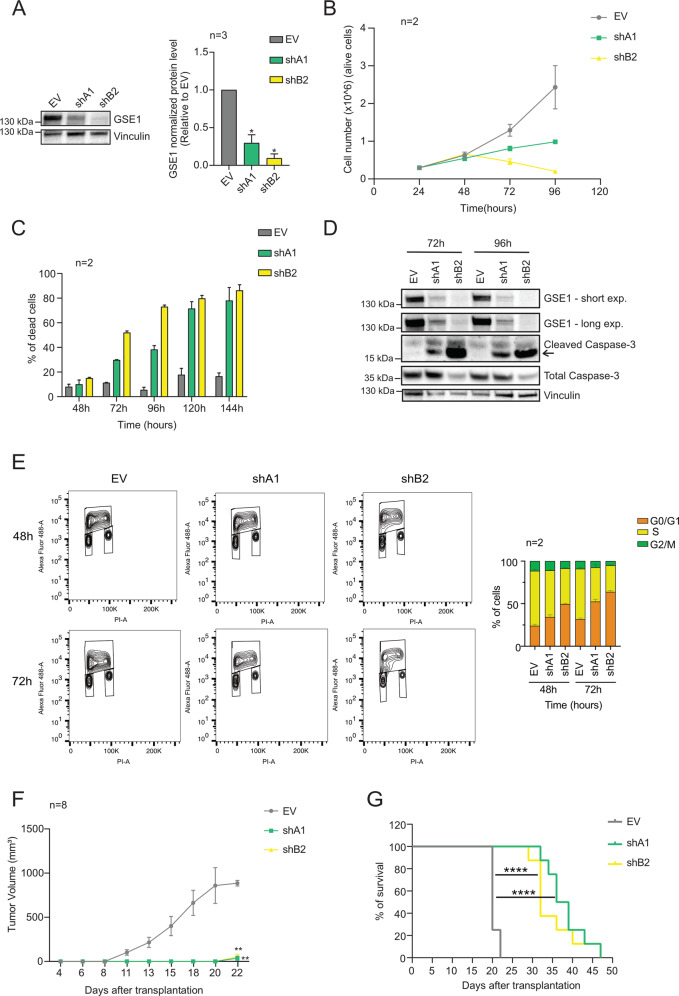
*GSE1* knockdown (KD) affects NB4 cell viability and in vivo tumor growth in NOD/SCID gamma (NSG) mice. **A** *Left panel:* Western blot analysis of GSE1 protein in NB4 cells transduced with either an empty lentiviral pLKO.1 puro vector (EV) or pLKO.1 puro containing the shRNA constructs targeting *GSE1* (shA1 and shB2) for 72 hours. Vinculin was used as loading control. *Right panel:* Bar graph displaying the quantitation of GSE1 protein level normalized over the Vinculin in three independent replicates (*n* = 3), whereby the results are plotted as fold change (FC) of GSE1 level in the knockdown (KD) samples over the control EV. The chart represents mean  ± SD (*n* = 3 biological replicates; one-sample T-test, *p value < 0.05). **B** Growth curve of NB4 cells transduced with either an empty pLKO.1 puro vector (EV) or pLKO.1 puro containing the shA1 and shB2 targeting *GSE1*. Graph represents mean ± SD from two biological replicates (*n* = 2). **C** Bar graph displaying the percentage of dead cells in control EV and *GSE1* KD cells. The analysis is performed after 48, 72, 96, 120, and 144 hours of infection. Cell viability was measured by trypan blue staining. The chart represents mean ± SD from two (*n* = 2) biological replicates. **D** Western blot analysis of GSE1, cleaved caspase-3, and total caspase-3 in cells transduced with either the control pLKO.1 puro (EV) or pLKO.1 containing the two shRNAs targeting *GSE1* (shA1 and shB2) for 72 and 96 h. Vinculin was used as loading control. The arrow indicates the cleaved form of the caspase-3. **E** *Left panel:* Contour-plot representation of multiparametric analysis, with propidium iodide (PI) at *x* axis and AlexaFluor®488 for BrdU at *y* axis, of BrdU incorporation in NB4 *GSE1* KD and control EV cells, at 48- and 72 h post infection. *Right panel:* Bar graph displaying the percentage of cells at each cell cycle phase, extrapolated from the BrdU incorporation assay. The chart represents mean ± SD from two (*n* = 2) biological replicates. **F** Tumor-growth curve in NSG mice transplanted subcutaneously with NB4 cells transduced 24 h earlier with either an empty pLKO.1 puro or pLKO.1 puro with the insert of the shRNAs (shA1 and shB2) targeting *GSE1* until day22 (when all mice transplanted with EV-transduced cells were dead). Graph represents mean ± SD of the tumor volume in each condition (*n* = 8, 8 distinct mice for each group; unpaired T-test, **p-value < 0.01 calculated at day 22). **G** Kaplan–Meier survival curve of mice transplanted with NB4 cells previously transduced with either an empty pLKO.1 vector or pLKO.1 containing shA1 or shB2 targeting *GSE1* (*n* = 8 for each group, log-rank Mantel–Cox test, ****p-value < 0.0001).

Next, we evaluated the phenotypic effects of *GSE1* downregulation in AML. To do it, we silenced *GSE1* in NB4 cells by RNA interference, using two short-hairpin RNAs (shRNAs) that displayed different efficiency in depleting *GSE1*, with shB2 being more effective than shA1 (Fig. 2A). We observed a robust reduction of cell viability, which was dependent on residual GSE1 protein level. Along with reduced cell growth, cell death measured by trypan blue staining also increased with time and correlated positively with silencing efficiency (Fig. 2B and C). This result was corroborated by the detection of cleaved caspase-3, a marker of apoptosis, in both shA1- and shB2- transduced cells 72 h post infection (Fig. 2D). The link between *GSE1* KD and apoptosis was further confirmed in shB2-transduced cells by measuring the reduction of total (not cleaved) caspase-3, which indicated that, in these cells, the majority of the enzyme was in its active form at 72 h post infection (Fig. 2D). Together, these experiments demonstrated that *GSE1* depletion impairs NB4 cell growth and induces apoptosis.

We also assessed the effect of *GSE1* downregulation on cell cycle progression by measuring both the DNA content of cells with propidium iodide (PI) (Fig. S5) and BrdU incorporation (Fig. 2E) following infection. We observed a 30% increase of cells in G0/G1-phase after *GSE1* KD, mirrored by a decrease of proliferating cells in S phase. Notably, shB2—which is more efficient than shA1 in downregulating *GSE1*—displayed stronger effects on cell cycle arrest in a time-dependent fashion.

Based on these results in vitro, we tested the effect of *GSE1* depletion on tumor growth in vivo: About 24 h post transduction with shA1, shB2, and control EV (Fig. S6A and S6B), NB4 cells were injected subcutaneously in NSG mice and tumor growth was measured at time intervals until mice were sacrificed when the tumor reached a maximal diameter of about 15 mm. *GSE1* KD strongly affected tumor growth, as indicated by the fact that mice injected with *GSE1* KD cells presented with palpable tumors only starting from day 22 after transplantation (Figs. 2F and S6C), when almost all control mice had already been sacrificed. WB analysis on the protein extracts from the tumoral masses confirmed that in 62% of cases (5 out of 8 tumors), the GSE1 depletion was maintained in the tumors grown from mice injected with *GSE1* KD cells, thus demonstrating that—in the majority of the mice—the cells with reduced GSE1 did not die in vivo and were able to develop tumors, even if more slowly compared with the control (Fig. S6D). Furthermore, the survival curve showed that mice transplanted with EV-transduced NB4 cells died between day 20 and day 22, whereas mice transplanted with *GSE1*-depleted cells had a prolonged lifespan, with a median survival time of 38 days (Fig. 2G). These in vivo results confirm the in vitro data and indicate that GSE1 sustains cellular oncogenic properties in AML.

### By reducing GSE1 protein levels, LSD1 inhibitors induce upregulation of differentiation-associated cytokine-mediated signaling and immune-response pathways in AML

**Fig. 3:**
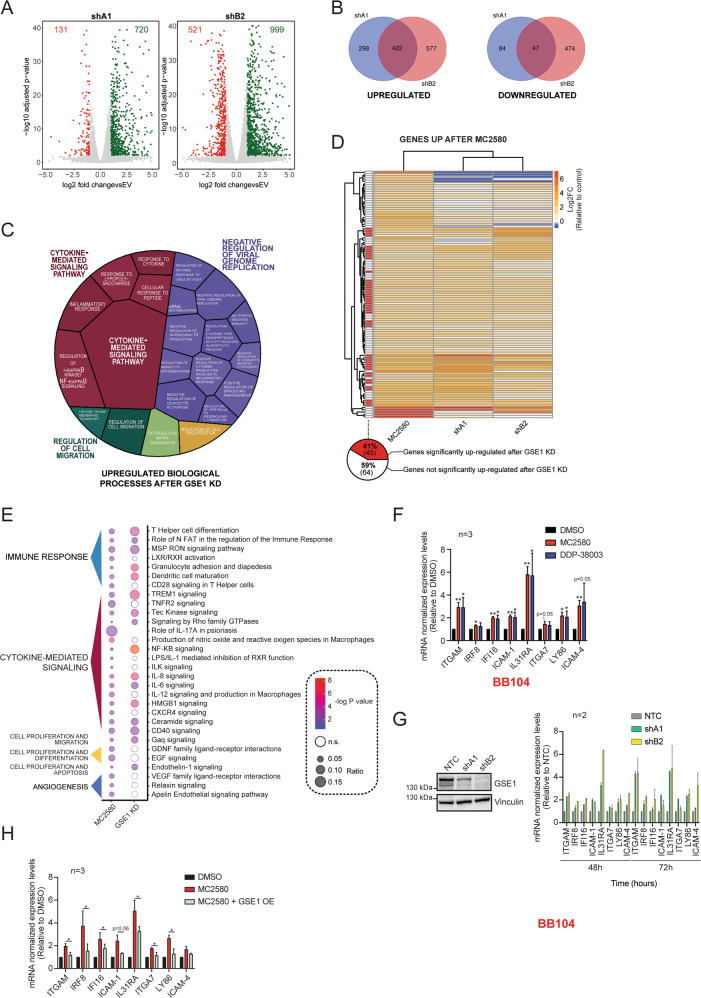
GSE1 downregulation elicited by LSD1 inhibitors induces transcription of genes associated with cytokine-mediated signaling and immune response pathways. **A** Volcano plot displaying up- and downregulated genes upon 48 h of transduction with pLKO.1 vector containing shA1 and shB2 inserts. The *x* axis shows the log_2_ fold-change (FC) values of each gene in the shRNAs-transduced cells compared with control empty vector (EV)-transduced, while the *y* axis displays the −log_10_-adjusted *p*-values (p-adj). FC value is calculated with DEseq2 program [59], using two biological replicates for each condition (*n* = 2). **B** Venn diagrams with the number of individual and overlapping upregulated and downregulated genes identified 48 h after transduction with shA1 and shB2 constructs. **C** Voronoi tree map displaying the statistically significant GO biological process (BP) terms associated with the common 422 upregulated genes upon *GSE1* KD with the two shRNA constructs. GO analysis and calculation of the statistically significant BP was performed through EnrichR [60] (adjusted *p*-value < 0.05), and then BP was grouped using Revigo [46]. Voronoi plot includes the BP terms mapped to a high hierarchical level. The tassel size corresponds to the −log_10_ *p*-value of the enrichment while the color intensity to the number of genes belonging to each category. **D** Unsupervised hierarchical clustering of all significantly upregulated genes upon MC2580 treatment (p-adj < 0.01, log_2_ FC > 1). Heatmap displays the log_2_ FC values of these genes in response to both *GSE1* KD and MC2580 treatment in NB4 cells. Each row in the heatmap represents an individual gene. Log_2_ FC values of each gene are plotted as the average of two independent biological replicates, for each condition (*n* = 2). Genes significantly upregulated by both shA1 and shB2 (p-adj < 0.01, log_2_ FC > 1) are flagged in red. Below is shown the pie-chart indicating the percentage of the 109 genes upregulated by MC2580 that are also significantly induced by *GSE1* KD with both shRNAs. **E** Bubble-plot displaying the statistically significant IPA pathways (−log_10_ *p*-value > 2) obtained from the analysis of the differentially expressed genes (DEG) after MC2580 treatment and comparison with the IPA results achieved from the DEG upon *GSE1* KD. The bubble size reflects the number of genes belonging to that pathway, while colors indicate the −log_10_ *p*-value associated with each IPA term. Nonsignificant IPA pathway terms are colored in white. IPA pathways are clustered manually, according to specific biological processes. Some IPA pathway terms are not displayed in the current figure panel, but the complete list is in Table S4. **F** RT-qPCR analysis on a panel of genes in *MLL-* translocated BB104 primary patient cells treated for 24 h with MC2580 (2 µM), DDP-38003 (2 µM) and DMSO as control. Ct values of different genes are normalized against *β-Actin*. The results are plotted as FC of the mRNA levels in the treated conditions compared with DMSO. The chart represents mean ± SD (*n* = 3 biological replicates, one sample T-test, **p-value < 0.01, *p-value < 0.05). Primers list in Table S7. **G** *Left panel:* Western blot analysis of GSE1 protein in *MLL-* translocated BB104 primary patient cells transduced with either a nontargeting shRNA construct (NTC) cloned in pLKO.1 puro vector or pLKO.1 puro containing the shRNA constructs targeting *GSE1* (shA1 and shB2) for 72 hours. Vinculin was used as loading control. *Right panel:* RT-qPCR analysis on a panel of genes at 48- and 72-h post transduction with shA1, shB2 and NTC as negative control in *MLL-* translocated BB104 primary patient cells. Ct values of the different genes are normalized over *β-Actin*. The results are plotted as FC of the mRNA levels in the *GSE1* KD samples, over the corresponding levels in the NTC-transduced cells. Bar graph represents mean ± SD (*n* = 2 biological replicates). Primer list in Table S7. **H** RT-qPCR analysis on a panel of genes upregulated by both LSD1 inhibitors and *GSE1* KD, in control and *GSE1*-over-expressing (OE) NB4 cells treated with 2 µM of MC2580 treatment. *GSE1* OE cells are generated by lentiviral transduction with the pLEX_307 vector containing the *GSE1* coding sequence (NM_001134473.3), while control cells are transduced with the empty pLEX_307 (pLEX_307 EV). Ct values for each gene are normalized against *GAPDH*. The results are plotted as FC of the mRNA levels in the treated conditions relative to the control DMSO. Bar graph represents mean ± SD from three (*n* = 3) biological replicates; paired T-test, *p-value < 0.05. Primer list in Table S7.

Next, we investigated the molecular mechanisms underpinning the phenotypic effects observed. Since GSE1 is a subunit in different transcriptional-regulatory complexes, we decided to assess the impact of *GSE1* depletion on the NB4 transcriptome by RNA-sequencing (RNA-seq) analysis of cells infected with either the two *GSE1*-shRNAs, or the EV as control. The analysis was carried out at 48 h post infection, a time point when cell death is still negligible (Fig. 2C). Upon *GSE1* KD, 720 genes were upregulated and 131 downregulated in shA1- infected cells, and 999 were upregulated and 521 downregulated genes in shB2-transduced NB4 (Fig. 3A, Table S1). This is in line with the evidence that GSE1 is mainly associated with corepressor complexes, such as the HDAC2 and BHC complexes [37, 39]. Intersecting the differentially expressed genes (DEG) in common between the two KD conditions, we obtained 422 common upregulated and 47 common downregulated genes (Fig. 3B). Gene Ontology (GO) analysis of the upregulated gene group revealed enrichment of terms related to immune and inflammatory responses and cell proliferation, while no significant enrichment of specific biological process occurred in the downregulated gene group (Table S2). In particular, using the Revigo Web server [46], we found the enrichment of the biological process (BP) terms “cytokine mediated signaling”, “negative regulation of viral genome replication”, “regulation of cell migration”, “extracellular matrix organization”, and “regulation of cell proliferation”. Within the term “negative regulation of viral genome replication”, we found biological processes associated with immune response (e.g., “neutrophil mediated immunity” and “negative regulation of leukocyte mediated cytotoxicity”), myeloid differentiation (e.g., “regulation of monocyte differentiation”), and apoptosis (e.g., “regulation of cysteine-type endopeptidase activity involved in apoptotic process”). Similarly, the term “cytokine mediated signaling” included several BP subterms linked to inflammation, such as “regulation of I − kappaB kinase/NF-kappaB signaling” (Fig. 3C). Similar results were obtained when we analyzed the upregulated gene sets with Reactome, to highlight the significant enriched biological pathways [47] (Fig. S7).

The transcriptomic data, indicating the upregulation of the general terms “negative regulation of viral genome replication” and “cytokine-mediated signaling” highlighted the role of this protein in regulation of biological processes such as myeloid differentiation, apoptosis, and proliferation.

In light of our observation that LSD1 pharmacological inhibition triggers GSE1 protein downregulation, we compared the transcriptomic changes observed upon *GSE1* depletion with the RNA-seq data of NB4 cells treated with MC2580, in order to unravel possible overlapping transcriptional programs that may suggest the presence of molecular pathways activated by LSD1 inhibitors that depend on GSE1. Compared with *GSE1* KD, MC2580 induced significantly fewer transcriptional changes, with only 109 significantly upregulated and 3 downregulated genes, respectively (Fig. S8, Table S3). This is in line with the stronger cellular effects observed upon *GSE1* downregulation compared with those elicited by LSD1 pharmacological inhibition. Under the hypothesis that a subset of genes may be transcriptionally induced by the drug through GSE1 protein downregulation, we first assessed the expression levels of the 109 genes upregulated by MC2580 within the *GSE1* KD cell transcriptome. Most genes modulated by MC2580 also showed an increasing trend upon *GSE1* depletion; in particular, 40% of them were significantly upregulated after infection with both shRNAs (Fig. 3D). Given this overlap, we assessed through Ingenuity Pathway Analysis (IPA) whether the molecular pathways activated by LSD1 inhibition were the same as those stimulated by *GSE1* depletion: 55% of the pathways triggered by the drug were also significantly activated by *GSE1* depletion and the majority were associated with the GO terms “cytokine-mediated signaling” and “immune response” (Fig. 3E, Table S4), which was included in the general-category GO term “negative regulation of viral genome replication” in Fig. 3C. We confirmed upregulation of some of the genes belonging to these biological processes by RT-qPCR upon MC2580/DDP-38003 treatment and *GSE1* KD in both NB4 (Fig. S9) and in cells from the *MLL-*translocated primary patient sample BB104 (Fig. 3F and G).

These results raised the possibility that LSD1 inhibitors upregulate mature myeloid genes associated with cytokine-mediated signaling and immune-response pathways via GSE1 downregulation, a hypothesis that was supported by profiling the expression of a panel of genes involved in these processes in NB4 cells upon MC2580 treatment, with or without *GSE1* overexpression. Observing that the upregulation of these genes upon LSD1 inhibition was significantly reduced when *GSE1* was over-expressed (Fig. 3H), we propose a novel function of GSE1 as an important corepressor of myeloid genes linked to inflammatory- and immune- response pathways and suggest the possibility of unlocking them through the targeting of *GSE1*.

### LSD1 pharmacological inhibition reduces GSE1 association with LSD1-bound promoters of genes involved in immune response and inflammatory pathways

Transcriptomic analysis suggested that the pharmacological inhibition of LSD1 leads activation of specific transcriptional programs contingent upon reduced protein levels of GSE1. Since GSE1 is a chromatin-associated factor, we decided to profile the effect of its reduction on chromatin by performing chromatin immuno-precipitation (ChIP). ChIP experiments were carried out in NB4 cells upon expression of the V5-tagged form of *GSE1*, whereby the V5 tag was used as bait for affinity enrichment of bound chromatin, to overcome the problem of the unavailability of ChIP-grade antibodies against GSE1. As the drug induces downregulation of the exogenous V5-GSE1 to a similar extent to the endogenous protein (Fig. S1), we reasoned that ChIP-seq analysis of the V5-tagged isoform could be a good proxy to assess the effects of LSD1 inhibitors on GSE1 genomic localization.

**Fig. 4:**
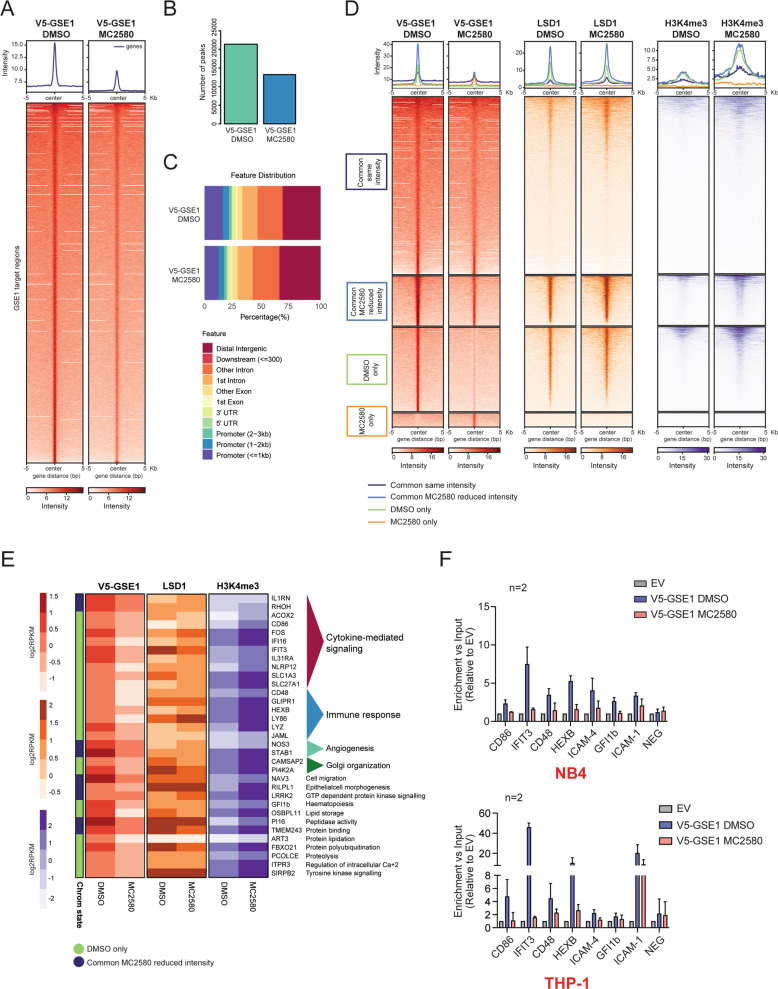
MC2580 decreases GSE1 localization on the LSD1-bound promoters of genes involved in cytokine-mediated signaling and immune-response pathways. **A** Heatmap representing the normalized V5-GSE1 ChIP-seq intensities ±5 kb around the center of the GSE1 target loci, at basal state and after 24 h of treatment with MC2580 (2 μM). V5-GSE1 ChIP-seq was performed in NB4 *GSE1* OE cells produced by lentiviral transduction of the pLEX_307 construct containing the *GSE1* coding sequence (NM_001134473.3) (pLEX_307 *GSE1*), while the negative control ChIP was carried out in cells transduced with the empty pLEX_307 (pLEX_307 EV). **B** Bar graph displaying the number of peaks extrapolated from the V5-GSE1 ChIP-seq in DMSO and MC2580-treated cells. **C** Genome annotation of the V5-GSE1 ChIP-seq peaks in control DMSO and LSD1-inhibited NB4 *GSE1* OE cells. **D** Heatmap displaying the normalized V5-GSE1, LSD1, and H3K4me3 ChIP-seq intensities ±5 kb around the center of the GSE1 target loci after 24 h of treatment with MC2580 or DMSO as control. The heatmap is grouped in different subgroups, according to the presence or absence or differential intensity of the GSE1 binding regions in the two conditions. The subset of “Common GSE1 targets with reduced intensity in DMSO” contains only 32 regions and is not displayed in the figure. The LSD1 and H3K4me3 ChIP-seq data used to generate the heatmap were published in ref. [13]. **E** Heatmap displaying the V5-GSE1, LSD1, and H3K4me3 ChIP-seq intensities at the promoters of genes upregulated after LSD1 inhibition, which show reduced binding of V5-GSE1 in MC2580-treated cells. Each row in the heatmap represents an individual gene. For the V5-GSE1 ChIP-seq, read intensities in regions identified as peaks and annotated to each gene are plotted as log_2_ RPKM. Instead, for the LSD1 and H3K4me3 ChIP-seq, read coverage (log_2_ RPKM) at promoter (±2.5 kb distance from the TSS) was represented. For each gene, its chromatin state is shown as referred in Fig. 4D, F) V5-GSE1 ChIP-qPCR profiling of a panel of GSE1-bound promoters of genes significantly upregulated by LSD1 inhibition in DMSO and MC2580 (2 μM)-treated NB4 (*upper panel*) and THP-1 (*lower panel*) cells overexpressing *GSE1* by pLEX_307 *GSE1* construct transduction. V5 ChIP in cells transduced with the pLEX_307 EV was used as negative control ChIP. NEG indicates a negative control region. Data are normalized over the respective input and displayed as fold change (FC) over the control EV. Bar graph represents mean ±standard deviation (SD) from two (*n* = 2) biological replicates. Primers list in Table S7.

ChIP-seq analysis proved that GSE1 binding to chromatin was globally reduced upon drug treatment, with the number and overall intensity of assigned peaks almost halved in treated cells (Fig. 4A and B). By inspection of the genomic distribution of the assigned peaks in both functional states, we found that at basal state GSE1 mainly localizes at distal intergenic (~25%), intronic (~35%), and promoter (~25%) regions, while upon LSD1 inhibition, its binding slightly increases at distal intergenic and intronic regions and decreases at the promoters (Fig. 4C).

We then grouped the genomic regions bound by GSE1 in four different categories: “Regions bound by GSE1 similarly in both DMSO- and MC2580-treated cells”, “GSE1- bound regions that display reduced binding upon MC2580”, “Regions bound by GSE1 only in DMSO condition” and “GSE1- bound regions only upon MC2580” (Table S5). We profiled by ChIP-seq the localization of LSD1 and different histone post translational modifications (PTMs) at these four genomic regions and found that LSD1 and all histone PTMs analyzed colocalized with V5-GSE1 in all genomic regions, except those bound exclusively by GSE1 upon drug treatment. Furthermore, LSD1 binding at GSE1 regions was not affected by the inhibitor, while histone PTMs associated with active transcription (mainly H3K4me3 and slightly H3K4me2) were enriched, particularly at those loci where GSE1 binding was reduced/abolished by the compound (“GSE1- bound regions that display reduced binding upon MC2580” and “Regions bound by GSE1 only in DMSO condition”) (Figs. 4D and S10). This suggests that the decreased association of GSE1 with chromatin may be linked to a chromatin state more prone to transcriptional activation. Moreover, considering the reduced localization of GSE1 at promoters upon MC2580 and the increased H3K4me3 level observed at the same regions (Figs. 4D and S11), we hypothesized that the decreased GSE1 association could directly mediate the transcriptional changes measured upon LSD1 inhibition. To confirm this hypothesis, we assessed the presence of promoters of the genes upregulated by MC2580 within the subsets of genomic regions where GSE1 binding was diminished by the drug and found several promoters of genes associated with cytokine-mediated signaling and immune response, like *IFI16*, *IL31RA*, *CD86*, and *CD48*. LSD1 co-localized with GSE1 at these promoters and its binding was not altered by the drug, while the H3K4me3 level increased (Fig. 4E). We validated the ChIP-seq results by ChIP-qPCR for some of these promoters in both NB4 and THP-1 cells. With ChIP-qPCR we also detected a reduced binding of GSE1 at the promoters of *ICAM-1* and *ICAM-4* upon MC2580, two other interesting genes involved in cytokine signaling pathways that were upregulated by LSD1 inhibition (Fig. 4F). The ChIP data corroborate the transcriptomic results and provide mechanistic insight into the effect of LSD1 inhibitors on GSE1 localization and activity at specific chromatin regulatory regions.

### GSE1 downregulation induced by LSD1 inhibition triggers myeloid differentiation

**Fig. 5:**
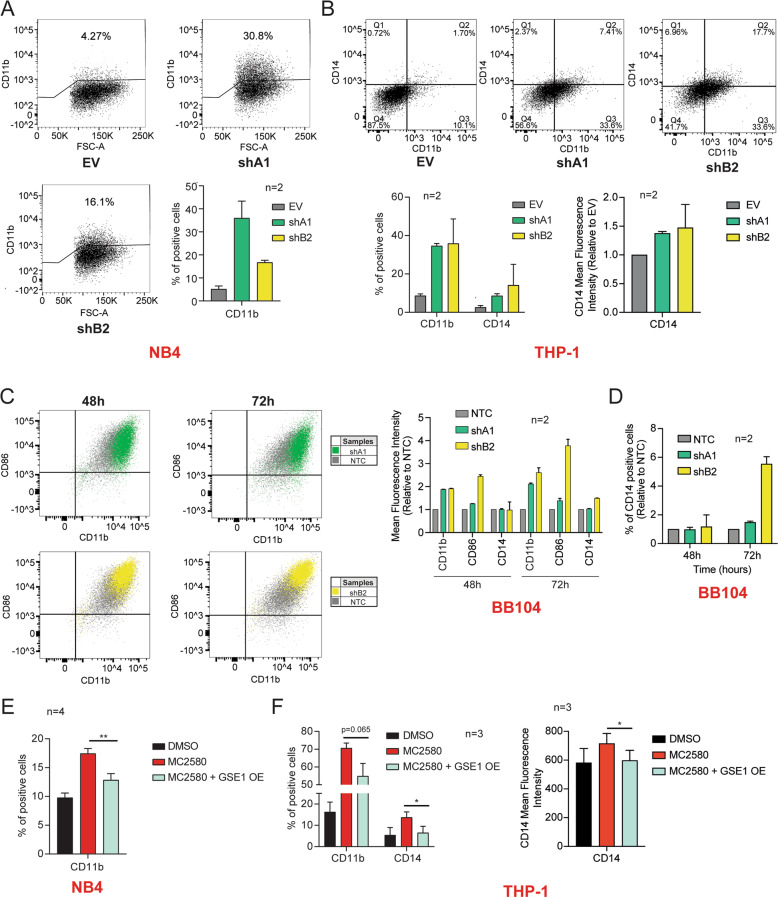
GSE1 downregulation contributes to the myeloid differentiation reactivation enforced by LSD1 inhibition. **A** Percentage of NB4 CD11b-positive cells measured by flow cytometry 48 h post transduction with the shA1 and shB2 cloned in pLKO.1 puro, or with the empty pLKO.1 puro vector (EV) used as negative control. Dot plots show the results obtained in one of the two biological replicates of the experiment. The bar chart represents mean  ± SD (*n* = 2 biological replicates). **B** *Left panel:* Percentage of CD11b- and CD14- positive THP-1 cells, assessed by flow cytometry analysis 48 h upon transduction with pLKO.1 puro vector containing shA1, shB2 or EV as negative control. Dot plots display the data of one of the two biological replicates of the experiment. The bar chart represents the mean + SD from two (*n* = 2) biological replicates. *Right panel:* Bar graph of the fold change (FC) of CD14 mean fluorescence intensity in THP-1 cells transduced for 48 h with shA1 and shB2, over the corresponding level in the EV-transduced cells. The chart represents mean + SD (*n* = 2 biological replicates). **C** *Left panel:* Representative dot plots showing the levels of CD11b- and CD86- positive cells as well as the intensity of these markers 48 h and 72 h post transduction with the shA1 and shB2 constructs targeting *GSE1,* or a nonspecific shRNA construct (NTC) cloned in pLKO.1 puro vector as negative control in the BB104 *MLL-* translocated primary patient sample. *Right panel:* Bar graph displaying the mean fluorescence intensity of CD11b, CD86 and CD14 in BB104 primary patient cells transduced for 48 h and 72 h with NTC, shA1 and shB2. The results are plotted as FC of the mean fluorescence intensity of these markers in the *GSE1* KD samples over the corresponding levels in the NTC-transduced cells. The chart represents mean ± SD (*n* = 2 biological replicates). **D** Percentage of BB104 CD14-positive cells measured by flow cytometry at 48- and 72-h post transduction with the shA1 and shB2 cloned in pLKO.1 puro, or with the NTC used as negative control. Data are plotted as FC of the CD14 mean fluorescence intensity in the *GSE1* KD samples over the corresponding levels in the NTC-infected cells. The chart represents mean ± SD (*n* = 2 biological replicates). **E** Percentage of CD11b-positive cells evaluated by flow cytometry in control (EV) and NB4 *GSE1* OE cells, treated for 24 h with MC2580 (2 μM). NB4 *GSE1* OE is produced by transducing the pLEX_307 vector with the *GSE1* coding sequence (NM_001134473.3), while control cells are transduced with the pLEX_307 EV. The chart represents mean ± SD (*n* = 4 biological replicates; paired T-test, **p-value < 0.01). **F** *Left panel:* Flow cytometry analysis of the percentage of CD11b- and CD14- positive cells in EV and *GSE1* OE THP-1 cells treated for 24 hours with MC2580. THP-1 *GSE1* OE is generated by lentiviral transduction of the pLEX_307 vector including *GSE1* coding sequence (NM_001134473.3), while control cells are transduced with the pLEX_307 EV. The chart represents mean ± SD (*n* = 3 biological replicates; paired T-test, *p-value < 0.05). *Right panel:* Bar graph displaying the CD14 mean fluorescence intensity in DMSO, MC2580 and MC2580 + *GSE1* OE THP-1 cells. The chart represents mean ± SD from three (*n* = 3) biological replicates (paired T-test, *p-value < 0.05).

Pharmacological inhibition of LSD1 represents a promising epigenetic approach for AML treatment through the release of the differentiation block and the induction of differentiation processes in leukemic blast cells [12, 19]. In line with this, our transcriptomic data demonstrated that the majority of the DEGs induced both by MC2580 and *GSE1* depletion were involved in hematological system development and hematopoiesis (Fig. S12). Thus, we hypothesized that the LSD1-dependent reduction of GSE1 on chromatin might be phenotypically linked to the induction of myeloid differentiation. To test this hypothesis, first we profiled by flow cytometry the expression of the cell surface differentiation marker CD11b in *GSE1* KD NB4 cells, observing 4- and 8- fold increase in the percentage of cells expressing this marker 48 hours post transduction with shB2 and shA1 constructs, respectively (Fig. 5A). Induction of CD11b upon *GSE1* KD was detected also in THP-1 monocytic cells, where we could also assess CD14, the monocyte differentiation antigen typically upregulated upon a differentiation stimulus. A 4- to 6- fold increase of CD14 was observed in *GSE1* KD cells compared with the control, together with about 40% increase of the mean fluorescence intensity. Furthermore, the majority of CD14- positive cells were also positive for CD11b (Fig. 5B). The higher concordance of the two shRNAs in inducing CD11b expression in THP-1 compared with NB4 cells could be partially explained by the observation that THP-1 responded more physiologically to *GSE1* KD. Indeed, no apoptosis was observed in THP-1 cell line 72 hours post transduction, with higher viability at early time points upon infection (Fig. S13). On the contrary, the elevated apoptotic process triggered by shB2 in NB4 cells could interfere negatively with the completion of the differentiation process [48, 49].

We also evaluated the level of the myeloid-differentiation markers CD11b, CD86 and CD14 in response to *GSE1* KD in the BB104 *MLL-*translocated primary patient sample. Despite already exhibiting high baseline expression, levels of CD11b and CD86 were further increased upon *GSE1* depletion with both shRNA constructs (Fig. 5C). For CD14, we detected an increase of the percentage of cells expressing this marker, especially upon shB2 transduction, at 72 h post infection. Furthermore, shB2 transduced cells also displayed at the same time point an increase in the CD14 mean fluorescence intensity (Fig. 5C, D).

Next, we asked whether GSE1 reduction induced by LSD1 inhibition was important to trigger myeloid differentiation by measuring with flow cytometry CD11b expression at 24 h post MC2580 treatment, in wild type (EV-infected) NB4 cells with and without GSE1 over-expression (OE). The observation that the increased CD11b level upon LSD1 inhibition was significantly reduced when GSE1 was overexpressed suggests that the differentiation process elicited by MC2580 also depends on GSE1 protein level (Fig. 5E). We also analyzed the expression of CD11b and CD14 in control (EV) THP-1 cells and in GSE1 overexpressing ones and confirmed the same results observed in NB4 (Fig. 5F). Interestingly, the overexpression of GSE1 in NB4 also led to an increase of the subpopulation of cells expressing the stem/progenitor marker c-KIT. This indicates that GSE1 not only prevents myeloid differentiation but also favors the maintenance of a stem-like immunophenotype, at least in this cell line (Figs. S14 and S15).

Altogether, the data collected led to the elaboration of a model whereby the inhibition of LSD1 with MC2580 and DDP-38003 reduces GSE1 protein level, leading to its reduced binding to chromatin and, in particular, to LSD1-target promoter regions regulating the expression of genes linked with cytokine-signaling and immune-response pathways, as well as of some transcription factors (TFs) important for myeloid differentiation, such as GFI1B. The consequent upregulation of these transcriptional programs enforces myeloid differentiation in leukemic cells.

### GSE1 displays oncogenic properties in patients with AML

The experimental evidence collected in this study suggests that GSE1 has protumorigenic activity in AML cells and that its downregulation can relieve the differentiation block in AML blast cells, suggesting this gene as a putative novel oncogene in this tumor type. To confirm the oncogenic properties of GSE1 in AML, we interrogated two large databases including clinical-pathological and RNA-seq data on 200 (AML-TCGA dataset) [50] and 535 AML patients (Tyner *et al*. dataset) [51], respectively.

**Fig. 6:**
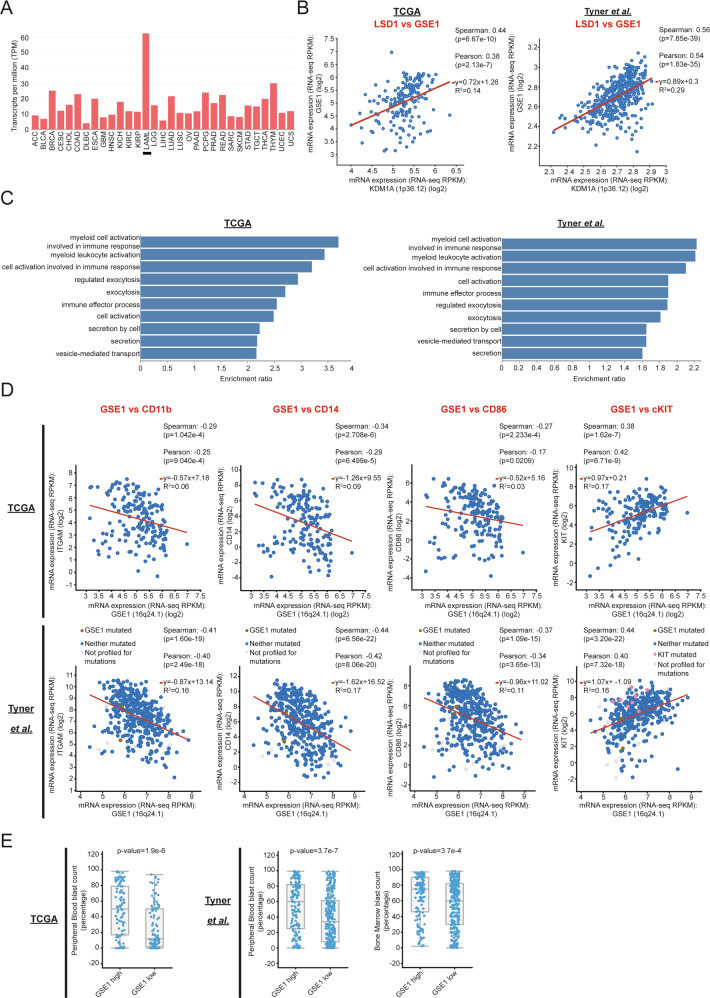
*GSE1* expression shows significant correlation with oncogenic features in AML patient samples. **A** Histogram showing the expression levels of *GSE1* mRNA across 31 different tumor histotypes from TCGA database. The *x* axis shows the tumor histotypes while the *y* axis displays the *GSE1* transcripts per million (TPM). **B** Scatter plots in logarithmic scale of relative *KDM1A* (on *x* axis) and *GSE1* (on *y* axis) mRNA expression levels, assessed in TCGA [50] (*left panel*) and Tyner et al. [51]. datasets (*right panel*). Red lines represent the best fit. Pearson and Spearman’s rank correlation coefficients, the equation of the line of best fit and the coefficient of determination (*R*^2^) are reported in the right corner of each panel. **C** GO analysis of the enriched biological processes (BP) on the list of genes whose expression levels are significantly negatively correlated with those of *GSE1* in the TCGA (*left panel*) and Tyner et al. datasets (*right panel*). Bar graphs represent the top10 enriched BP ranked according to the enriched ratio. The *y* axes represent the BP terms while the *x* axes the enrichment ratio. **D** Scatterplots in logarithmic scale of relative *ITGAM* (*1*^*st*^ *panel from the left*; on *y* axis), *CD14* (*2*^*nd*^ *panel from the left*; on *y* axis), *CD86* (*3*^*rd*^ *panel from the left*; on *y* axis), *c-KIT* (*4*^*th*^ *panel from the left*; on *y* axis) and *GSE1* (on *x* axes) mRNA expression levels, assessed in TCGA (*upper raw*) and Tyner et al. datasets (*lower row*). Red lines represent the best fit. Pearson and Spearman’s rank correlation coefficients, the equation of the line of best fit and the coefficient of determination (R^2^) are reported in each panel. **E** Box and whisker plots showing on the *y* axes the percentage of peripheral blood (*1*^*st*^ *panel and 2*^*nd*^ *panel from the left*) and bone marrow (*3*^*rd*^ *panel from the left)* blast count of AML patients grouped on *x* axes according to low (i.e., below median value; *GSE1*-low) and high (i.e., above median value; *GSE1*-high) *GSE1* mRNA tumor expression levels. Data from TCGA and Tyner et al. datasets are represented in the *1*^*st*^ *panel and in the 2*^*nd*^ *and 3*^*rd*^ *panels*, respectively.

First, we assessed the expression of *GSE1* across 31 different tumor histotypes available in the TCGA and found that AML is characterized by the highest *GSE1* expression (Fig. 6A) supporting a relevant role in AML pathogenesis. *GSE1* expression positively correlates with that of *KDM1A* (LSD1), both in AML-TCGA (*r*_s_ = 0.44; *p*-value = 6.7e-10) and Tyner et al*.* datasets (*r*_s_ = 0.56, *p*-value = 7.8e-39) (Fig. 6B).

Since our in vitro data showed that GSE1 acts as a corepressor of genes linked to inflammatory, immune-response and myeloid-differentiation pathways, we analysed the RNA-seq profiles from both datasets to identify genes whose expression levels negatively correlate with *GSE1*. Indeed, we found that the expression of 2157 and 5649 genes negatively correlated with that of *GSE1* (FDR < 0.05) in the AML-TCGA and Tyner et al. datasets, respectively (Table S6). GO analysis of these genes confirmed a significant enrichment (FDR < 0.05) of terms related to inflammatory- and immune- response pathways, as well as of pathways associated with myeloid differentiation (Fig. 6C). A sensitivity analysis conducted only on genes that displayed a stronger negative correlation with *GSE1* (i.e., *r* < −0.5 and FDR < 0.05; 239 genes in Tyner et al*.* and 38 genes in TCGA dataset, respectively) confirmed the same results (Fig. S16).

Moreover, we also detected a significant negative correlation between *GSE1* level and the expression levels of three myeloid-differentiation markers in AML patients: *ITGAM* (CD11b) (*r*_s_ = −0.29, *p*-value = 1.04e-4 in TCGA; *r*_s_ = −0.41, *p*-value = 1.6e-19 in Tyner et al.), *CD14 (r*_s_ = −0.34, *p*-value=2.7e-6 in TCGA; *r*_s_ = −0.44, *p*-value = 6.5e-22 in Tyner et al*.*) and *CD86* (*r*_s_ = −0.27, *p*-value = 2.23e-4 in TCGA; *r*_s_ = −0.37, *p*-value = 1.09e-15 in Tyner et al*.*), as well as a positive correlation with the stem/progenitor marker *c-KIT* (*r*_s_ = 0.38, *p*-value = 1.62e-7 in TCGA; *r*_s_ = 0.44, *p*-value = 3.20e-22 in Tyner et al*.*) (Fig. 6D). Altogether, these results confirm the transcriptomics and flow cytometry data acquired in AML cell lines, corroborating our model.

To further confirm the negative role of *GSE1* in myeloid differentiation processes, we evaluated the association between its expression levels and patients’ blasts percentage, a marker of degree of impaired myeloid differentiation, measured in both peripheral blood (PB) and bone marrow (BM). In both datasets, the group of patients with tumors expressing high *GSE1* levels (namely, *GSE1* expression level above the median value) had a significantly higher PB blast percentage compared with AML patients expressing low *GSE1* (i.e., *GSE1* expression level below the median value): the median blast percentage value in the *GSE1*-high versus the *GSE1*-low group was, respectively: 50% [IQR: 17–79%] versus 12% [IQR:2–50%] in TCGA dataset (*p*-value: 1.9e-6), and 60% [IQR: 25–82%] versus 34% [IQR:8–61%] in Tyner et al*.* dataset (*p*-value:3.7e-7). Data on BM blast percentage were available only from the Tyner et al. dataset and confirmed higher blast percentage in AML patients with high *GSE1* levels: the median blast percentage was 75% [IQR: 46–90%] in *GSE1*-high versus 60% [IQR: 30–82%] in *GSE1*-low group (*p*-value: 3.7e-4) (Fig. 6E).

Finally, we explored the possible link between the overall survival (OS) of AML patients and *GSE1* expression levels, unbiasedly categorized as below and above its median value. In the overall population, there was no association between *GSE1* expression and patient prognosis in either of the two datasets. However, we also analyzed the subgroup of patients with the M3-AML subtype (APL) considering that most of our in vitro and in vivo experiments were carried out in the NB4 M3 cell line. The pooled analysis of 23 M3-AML patient samples from the two datasets revealed that patients expressing high *GSE1* level have a poorer OS compared with low *GSE1* ones, consistent with our hypothesis (median OS not reached in *GSE1*-low group versus 46 months in *GSE1*-high group; log-rank *p*-value:0.057). Notably, patients with *GSE1*-low tumors displayed an impressively good prognosis, with only 1 death reported in 12 patients, while about 50% of patients with *GSE1*-high tumors died (5 out of 11) (Fig. S17).

The data acquired from the AML patient datasets are overall in agreement with the results acquired in vitro and pave the way to more thorough studies on the role of GSE1 in the onset and development of AML. They also suggest novel therapeutic perspectives based on the targeting of this protein by LSD1 inhibitors to reactivate the myeloid differentiation process.

## Discussion

In this study, we show that the LSD1 inhibitors MC2580 and DDP-38003 can elicit myeloid differentiation in AML through downregulation of GSE1 protein, a poorly explored LSD1 interactor. Our data add another layer of information on the mechanism of action of these drugs in leukemia, beyond the already known inhibitory effects on the lysine histone demethylase activity [21] and on LSD1–GFI1 interaction [12, 13]. Furthermore, our results unveil the protumorigenic activity of GSE1, which has not previously been described in AML.

Our data suggest that the drugs in use most likely affect GSE1 translation rather than transcription or stability, even if the details of the mechanism remain to be elucidated. Based on the latest literature [32, 33, 52], the most probable mechanism underpinning GSE1 protein depletion involves the miRNA machinery; however, our preliminary, unpublished data seem to exclude it, while pointing toward the impairment of protein translation. The assembly of the translation initiation complex in particular is a very critical step of protein synthesis and the interference of this process has been observed often in association with cancer development [53]. Furthermore, our indications on translational inhibition are coherent with recent data showing that—in some cell lines—LSD1 inhibitors activate the mTOR-signaling cascade [54, 55], which directly affects protein synthesis [56]. Future investigations will be devoted to dissecting mechanistically how LSD1 inhibitors modulate GSE1 translation.

The RNA-seq data show that in NB4 cells *GSE1* depletion induces more extensive transcriptional changes than those caused by LSD1 inhibitors. In line with this, the phenotypic effects elicited upon *GSE1* KD are more pronounced than those observed upon treatment with LSD1 inhibitors, the latter of which consist in a mild reduction of cell viability and colony-forming ability in liquid and semi-solid culture, respectively [13]. These molecular and phenotypic differences can be explained considering the significantly different reduction of GSE1 protein level caused by these two perturbations, with MC2580/DDP-38003 leading to a milder GSE1 downregulation (around 40%) than the shA1 and shB2 constructs (around 80–90%). The dose-dependency of the phenotypic effects to *GSE1* reduction induced by the two shRNA constructs used in this study corroborates this hypothesis. The evidence that cellular levels of GSE1 are critical for NB4 cell viability is particularly intriguing, as it suggests that this gene has oncogenic function in AML and could represent a new target for treatment of this type of tumor.

From a molecular standpoint, the transcriptomic data collected in this study indicate that the induction of genes involved in cytokine-signaling and immune-response pathways by LSD1 inhibitors is—at least in part—GSE1-dependent. Specifically, as suggested by our ChIP-seq and RNA-seq data, we propose that this transcriptional response is mediated by the reduction of GSE1 on chromatin and, in particular, at LSD1-bound promoters. It should be noted, however, that the ChIP-seq data also showed binding of GSE1 to distal intergenic and intronic regions and overall colocalization with H3K4me1 and H3K27ac, two histone modifications traditionally associated with enhancers. This observation points towards the presence of GSE1 at other *cis*-regulatory elements, so that its reduced association to chromatin after drug treatment could cause more global transcriptional effects, still to be described.

The analysis of *GSE1* expression in AML patient samples and the correlation analysis with different oncogenic features, such as the BM and PB blast percentage, confirm the evidence collected from cellular models on the oncogenic properties of this gene in AML. Together with the finding that pharmacological inhibition of LSD1 reduces GSE1, these data suggest the possibility of stratifying AML patients based on *GSE1* expression levels to identify those who could benefit more from LSD1 inhibitor treatment.

In summary, by describing for the first time the molecular and cellular implications of GSE1 modulation in AML, this study paves the way for a thorough assessment of the role of this chromatin factor in hematological malignancies and the possibility of targeting it with LSD1 inhibitors, to trigger myeloid differentiation for treatment of this type of tumor.

## Materials and methods

### Cell culture

NB4 and THP-1, respectively human promyelocytic and monocytic leukemia-derived cell lines (M3 and M5) were grown in RPMI plus 10% of fetal bovine serum (FBS), 2 mM glutamine (Glu) and 1% penicillin/streptomycin (P/S). SKNO-1 human acute myeloblastic leukemia-derived cells (M2) were cultured in the same conditions, with the addition of 10 ng/ml GM-CSF. OCI-AML2 human acute myeloid leukemia-derived cells (M4) were grown in 80% alpha-MEM supplemented with 20% FBS, 2 mM Glu and 1% P/S. NB4, THP-1, SKNO-1, and OCI-AML2 cell lines were routinely tested for mycoplasma contamination by PCR method and MycoAlert (Lonza #LT07-118) kit at the IEO tissue culture facility. As previously described [12], BB104 *MLL-* translocated primary patient cells (with a variant *t*(9;11) translocation) were cocultured on MS5 stromal cells and grown in alpha-MEM medium supplemented with 12.5% heat-inactivated FBS, 12.5% heat-inactivated horse serum, 2 mM Glu, 1% P/S, 57.2 μM β-mercaptoethanol, 1 μM hydrocortisone, 20 ng/ml IL-3, 20 ng/ml G-CSF and 20 ng/ml TPO (Peprotech). Primary human AML samples were from Manchester Cancer Research Centre’s Tissue Biobank (Manchester, UK) and used with the informed consent of donors. The Biobank holds a generic ethics approval (18/NW/0092) which can be conferred to users of banked samples via the MCRC Biobank Access Policy. Samples used in this project were approved for use under application number 08_TISO-02. Cultures were maintained in a humidified tissue culture incubator at 37 °C in 5% CO_2_.

### Compounds

The LSD1 inhibitors MC2580 and DDP-38003 were provided by the Department of Drug Chemistry and Technologies of the Sapienza University of Rome (Italy) and the Experimental Therapeutic Unit of the IFOM-IEO Campus, respectively [27, 28]. Cycloheximide was purchased from Sigma Aldrich (C7698).

### RNA sequencing (RNA-seq) and data analysis

mRNA-sequencing (mRNA-seq) libraries were prepared with the TruSeq RNA Sample Preparation v2 kit (RS-122-2002, Illumina) according to the manufacturer’s protocol, starting from 500 ng of total RNA per sample. Sequencing was performed using the NovaSeq 6000 (Illumina) instrument. Raw reads were mapped to the human reference genome hg38 using STAR aligner [57] and quantified through the *rsem-calculate-expression* function of the RSEM package [58]. Differentially expressed genes (DEG) were determined with the DEseq2 package [59], as follows: genes with an adjusted *p*-value lower than 0.01 and a log_2_ fold change (FC) greater than 1 and smaller than −1 (log2 FC < −1 and >1) were considered as upregulated and downregulated, respectively. GO analysis of the enriched biological processes (BP) was carried out with the EnrichR software [60], while biological pathway analysis was executed using the Reactome database [47] contained within EnrichR software and the Ingenuity Pathway Analysis (IPA) (QIAGEN Inc., https://www.qiagenbioinformatics.com/products/ingenuitypathway-analysis). Significant BP and Reactome terms had an adjusted *p*-value < 0.05, while significant IPA pathways had a –log(*p*-value) >2. Voronoi plot of the GO was generated using the R-package voronoiTreemap (https://github.com/uRosConf/voronoiTreemap).

### Lentiviral and retroviral constructs for exogenous protein expression

NB4 LSD1 KO cells and NB4 LSD1 KO cells transduced with the N-terminal truncated (172–833) form of LSD1 or the empty PINCO vector were generated as previously described in [13]. To knockdown (KD) *GSE1*, short-hairpin RNA (shRNA) constructs targeting the protein were cloned into the pLKO.1 puro expression vector. The primers used for shRNA production and cloning were the following:

1. shA1:
  - FW 5′-CCGGGAACTCACCTTGACGTCAATGCTCGAGCATTGACGTCAAGGTGAGTTCTTTTTG-3′
  - REV 5′- AATTCAAAAAGAACTCACCTTGACGTCAATGCTCGAGCATTGACGTCAAGGTGAGTTC-3′
2. shB2:
  c. FW 5′-CCGGCTGAGCATGCTTCACTATATCCTCGAGGATATAGTGAAGCATGCTCAGTTTTTG-3′
  d. REV 5′- AATTCAAAAACTGAGCATGCTTCACTATATCCTCGAGGATATAGTGAAGCATGCTCAG-3′

In *GSE1* KD experiments using BB104 *MLL-*translocated primary patient cells, a nontargeting shRNA construct (NTC) with at least four base-pair mismatches from known human and murine genes was used as a negative control [12].

To overexpress *GSE1*, its coding sequence transcript (GenBankTM accession number NM_001134473.3) was cloned into the pLEX_307 (Addgene, #41392) and the pLEX_306 (Addgene, #41391) vector backbones, thus producing the pLEX_306 *GSE1* and the pLEX_307 *GSE1* constructs. *GSE1* coding sequence contained within the vector pENTR223 was first purchased by DNASU plasmid repository (HsCD00623069) [61]: the plasmid was a kind gift from David Hill and David Root, from the Dana–Farber Cancer Institute, Broad Institute of Harvard, and Massachusetts Institute of Technology (MIT), as a part of the ORFeome Collaboration [62]. Subsequently, *GSE1* coding sequence was modified using the Q5 Site-directed Mutagenesis Kit (E0554S, New England Biolabs) to substitute the cytosine (C) in position 2495 with a thymine (T), a necessary step to generate the coding sequence of the wild-type *GSE1* transcript variant 2 (NM_001134473.3). At this point, the coding sequence was inserted through the Gateway System Technology into the pLEX_306 and pLEX_307 vectors [63].

### In vivo studies

In vivo studies were performed after approval from our animal facility and the institutional welfare committee “Organismo Preposto al Benessere degli Animali (OPBA)”. Experiments were notified to the Ministry of Health (as required by the Italian law; Institutional Animal Care and Use Committee numbers: 71/2019 in accordance with European Union directive 2010/63). NB4 cells were transduced with the empty pLKO.1 puro (EV) or pLKO.1 puro containing the shA1 and the shB2. After 24 hours, 1.5 × 10^6^ cells per mouse were resuspended in 200 µl PBS containing 15% of Matrigel (Corning, 356231) and, then, injected subcutaneously in the left flank of 8–12-week old male and female NOD SCID IL2Rgnull (NSG) mice. For each mouse, the tumor size was measured three times per week with a linear caliper and the volume was calculated using the formula V = (a × b^2^)/2, where a and b are the longest and the shortest diameters of the tumor, respectively. The results are reported as tumor volume (mm^3^). Mice were sacrificed when the longest diameter of the tumor reached a size of approximately 15 mm.

### Flow cytometry analysis of cell-surface markers

About 1 × 10^6^ cells were harvested, resuspended in 300 µl of 5% BSA dissolved in PBS and blocked for 30 min at room temperature. Then, cells were repelleted and resuspended in 100 µl of primary antibody diluted in 1% BSA in PBS and let for 1 h at room temperature in the dark. At this point, cells were washed with 1 ml of 1% BSA in PBS, centrifuged and resuspended in 250 µl of cold PBS. Transduced cells were, then, fixed by the addition of 250 µl 2% formaldehyde in PBS and incubated for 20 min in ice. After a further spinning, cell pellets were resuspended in 500 µl of PBS and analyzed by the FACS Celesta flow cytometer (BD Biosciences). Data analysis was performed using the FlowJo software. The antibodies used for the flow cytometry analysis of the cell surface markers were: anti-human CD11b (740965, BD OptiBuild™, 1:100), anti-human CD14 (11-0149-42, Thermo Fisher Scientific, 1:100), and anti-human CD117 known also as c-KIT (12-1178-42, Thermo Fisher Scientific, 1:100). Flow cytometry analysis of *MLL-* translocated primary patient cells are carried out as described in [12] and the antibodies used were: anti-human CD11b (12-0118-42, Thermo Fisher Scientific, 1:200), anti-human CD86 (46-0869-42, Thermo Fisher Scientific, 1:200), and anti-human CD14 (46-0149-42, Thermo Fisher Scientific, 1:200).

### Chromatin immuno-precipitation-sequencing (ChIP-seq)

ChIP-seq analysis was carried out in NB4 cells transduced with the pLEX_307 *GSE1* and treated for 24 hours with either MC2580 or DMSO. As negative ChIP control, we used NB4 cells transduced with the empty vector pLEX_307 (pLEX_307 EV). About 1 × 10^8^ cells for each condition were cross-linked by formaldehyde at 1% final concentration, which was added to the culture medium and incubated for 10 min with shaking. The reaction was stopped with 0.125 M glycine and then samples were left shaking for 5 min at room temperature. Cells were then washed twice with PBS and lysed in SDS buffer (50 mM Tris-HCl pH 8.0, 0.5% SDS, 100 mM NaCl, 5 mM EDTA, 0.02% NaN_3_), supplemented with protease inhibitors (Roche, 04693116001) and 0.5 mM PMSF. At this point, we added the Triton Dilution buffer (100 mM NaCl, 100 mM Tris-HCl pH 8.5, 5 mM EDTA, 5% Triton X-100, 0.02% NaN_3_) supplemented with protease inhibitors (Roche, 04693116001) and 0.5 mM PMSF to the whole cell extracts to obtain the IP buffer conditions (100 mM NaCl, 33 mM Tris-HCl pH 8.0, 5 mM EDTA, 0.02% NaN_3_, 0.33% SDS, 1.7% Triton X-100, 33 mM Tris-HCl pH 8.5). Chromatin was then subjected to 35 cycles of sonication (30 seconds each) using a Branson Sonifier 250 to obtain DNA fragments of 300-bp average length. Subsequently, sheared chromatin was precleared by incubation with 100 µl of Dynabeads protein G (Invitrogen, 10004D) for 2 hours on a rotating wheel at 4 °C. After preclearing, chromatin was used as input in the immuno-precipitation experiment, carried out overnight on a rotating wheel at 4 °C in the presence of 20 µg of anti-V5 antibody (Abcam, ab9116). About 2.5% of the input sample was collected just before the addition of the antibody and stored at −20 °C, for subsequent tests. After the overnight incubation, 200 µl of Dynabeads protein G were added to the antibody–chromatin reaction tube and incubated for 3 hours on a rotating wheel at 4 °C. Later, beads were washed thrice with Washing buffer A (1% Triton X-100, 0.1% SDS, 150 mM NaCl, 2 mM EDTA, pH 8, and 20 mM Tris-HCl, pH 8) and once with Washing buffer B (1% Triton X-100, 0.1% SDS, 500 mM NaCl, 2 mM EDTA, pH 8, and 20 mM Tris-HCl, pH 8) supplemented with protease inhibitors (Roche, 04693116001), followed by a final washing step with TE 1X buffer. At each wash, beads were incubated for 5 min on a rotating wheel at 4 °C. De-cross-linking/elution step of the ChIP samples was carried out by the addition of 300 µl of de-cross-linking buffer (2% SDS, 0.25 mg/ml Proteinase K in TE 1X), followed by overnight incubation at 65 °C. The de-crosslinking step was also carried out for the input sample, by adding three volumes of de-crosslinking buffer to 1 volume of input. The day after, DNA from ChIPs and input samples was purified using the DNA purification kit (QIAquick PCR Purification Kit, Qiagen, 28106) according to the manufacturer’s protocol. DNA libraries were prepared with 10 ng of DNA through an in-house protocol [64] by the IEO genomic facility and sequenced on a NovaSeq 6000 (Illumina) instrument.

### ChIP-seq data analysis

Short reads obtained from Illumina Genome Analyzer II were quality-filtered according to the ENCODE pipeline [65]. Reads were aligned to the hg38 reference genome using Bowtie (v 4.8.2) [66]. MACS (v 1.4.2) [67] was used as peakcaller to identify regions of ChIP-seq enrichment of the V5-GSE1 in DMSO and MC2580-treated cells, using as background both the respective input and the V5-ChIP performed in NB4 cells transduced with the pLEX_307 EV. The resulting enriched regions were annotated as specifically bound by GSE1 in each condition. Only reads with a unique match to the genome and with two or fewer mismatches (-m 1 –v 2) were retained. MACS *p*-value threshold was set to 10^−5^ for all the data sets. The four genomic region categories displayed in Fig. 4D and Fig. S10-S11 were obtained as follows: genomic regions bound by GSE1 in both DMSO- and MC2580-treated cells were defined as regions with peaks in both conditions and at least 1 bp of overlap between the two samples. These loci were further divided in “common same intensity”, which displayed a log_2_FC RPKM of DMSO vs MC2580 < 0.5 and “common MC2580 reduced intensity” showing a log_2_FC RPKM of DMSO vs MC2580 > 0.5. Regions commonly detected in both conditions and showing a log_2_FC RPKM of MC2580 vs DMSO > 0.5 were only 32 (“common DMSO reduced intensity”) and were not shown in the figures. “DMSO only” and “MC2580 only” categories instead contained genomic regions with identified peaks in DMSO or MC2580-treated cells, respectively. Within these regions, read counts were calculated with bedtools suite and only regions with a log_2_FC RPKM of DMSO vs MC2580 > 1 for “DMSO only” and log_2_FC RPKM of MC2580 vs DMSO > 1 for “MC2580 only” were kept. Annotation of the genomic regions in the different conditions was achieved using the R package ChIPseeker [68] and ChIPpeakAnno [69]. The bigwig files for UCSC browser visualization of genome profiles were normalized with the deepTools suite. ChIP-seq data of LSD1, H3K4me1, H3K4me2, H3K4me3 and H3K27ac used for comparative analysis with V5-GSE1 ChIP-seq are described and present in [13].

### Analysis of GSE1 expression in large datasets with AML patient samples and its correlation with some oncogenic properties

The analysis was performed using two datasets, containing both clinical–pathological and RNA-seq data of tumor samples from patients with AML [50, 51]. Data were retrieved from cBioPortal repository (https://www.cbioportal.org) [70]. Details on the clinical–pathological characteristics of the two cohorts of patients have been previously reported [50, 51], as well as the extensive procedures adopted by cBioPortal repository for reprocessing and normalizing expression data, quality control assessment and standardization of the datasets to maximize comparability [70]. Correlation analysis was performed to assess the association between the expression of *GSE1* and *KDM1A* (LSD1) as well as selected genes known to be differentiation or stem/progenitor markers (i.e., *ITGAM, CD14, CD86, c-KIT*). The results were expressed in terms of Pearson and Spearman’s rank correlation coefficients, the equation of the line of best fit and the coefficient of determination (R^2^). Correlation analysis was also performed on the whole tumor transcriptome to identify all genes whose expression levels were negatively and significantly (i.e., FDR < 0.05) correlated with those of *GSE1*. GO analysis of the enriched biological processes (BP) was carried out on the list of genes found negatively and significantly correlated with *GSE1*, with the WebGestalt (WEB-based Gene SeT AnaLysis Toolkit) tool [71]. Significantly enriched BPs had an adjusted *p*-value < 0.05. The top 10 significantly enriched BP terms, ranked according to the enriched ratio, were reported. A sensitivity analysis conducted only on genes whose expression levels were strongly negatively (i.e., r < −0.5) and significantly (i.e., FDR < 0.05) correlated with those of *GSE1* was also performed. Finally, survival analysis of AML patients from the two cohorts was performed applying the Kaplan–Meier method. Patients were unbiasedly grouped according to the tumor expression levels of *GSE1* (i.e., above and below its median expression value), and log-rank test was used to compare the survival distributions of the two patients’ groups. Subgroup analysis was performed to compare survival distributions of patients with the M3-AML subtype grouped according to *GSE1* tumor levels. Considering the limited number of patients with such AML subtype, survival analysis was carried out pooling patients from the two datasets.

## Supplementary information

supplementary material

Table S1

Table S2

Table S3

Table S4

Table S5

Table S6

Table S7

## Data Availability

The accession number for all the RNA-seq data and the V5-GSE1 ChIP-seq data reported in this paper is GEO database: GSE164560. The accession number for the LSD1, H3K4me1, H3K4me2, H3K4me3 and H3K27ac ChIP-seq data reported in ref. [13] is GEO database: GSE128530. The accession number for the MS-proteomics data reported in ref. [13] is ProteomeXchange database: PXD012954.
