# Supplementary material for: Pharmacological inhibition of LSD1 triggers myeloid differentiation by targeting GSE1 oncogenic functions in AML
Source: Oncogene. 2021 Dec 3;41(6):878–94. doi: 10.1038/s41388-021-02123-7 (PMC8830420; doi:10.1038/s41388-021-02123-7)
Supplement: Supplementary file 1 — supplementary material [file 41388_2021_2123_MOESM1_ESM.docx]

**Supplementary Materials for**

**Pharmacological inhibition of LSD1 triggers myeloid differentiation by targeting GSE1 oncogenic functions in AML**

Luciano Nicosia^1,#,+^, Francesca Ludovica Boffo^1,+^, Elena Ceccacci^1^, Fabio Conforti^1^, Isabella Pallavicini^1^, Fabio Bedin^1^, Roberto Ravasio^1^, Enrico Massignani^1^, Tim C. P. Somervaille^2^, Saverio Minucci^1,3^ and Tiziana Bonaldi^1,4 *^

^1^ Department of Experimental Oncology, IEO, European Institute of Oncology IRCCS, Milan 20139, Italy.

^2^ Leukaemia Biology Laboratory, Cancer Research UK Manchester Institute, The University of Manchester, Oglesby Cancer Research Centre Building, Manchester, M20 4GJ, United Kingdom (UK).

^3^ Department of Biosciences, University of Milan, Milan 20133, Italy.

^4^ Department of Oncology and Haemato-Oncology, University of Milan, Milan 20133, Italy

^#^ Current address: Leukaemia Biology Laboratory, Cancer Research UK Manchester Institute, The University of Manchester, Oglesby Cancer Research Centre Building, Manchester, M20 4GJ, United Kingdom (UK).

^+^ These authors contributed equally to this work.

^*^ Corresponding author: [tiziana.bonaldi@ieo.it](mailto:tiziana.bonaldi@ieo.it), tiziana.bnaldi@unimi.it

The PDF file contains: Supplementary Materials and Methods, Supplementary Figures from S1 to S17 with the corresponding legends, legends of the Table S1, S2, S3, S4, S5, S6 and S7 (these tables are also included as Supplementary Materials), References.

**Supplementary Materials and Methods**

**Protein co-immunoprecipitation (co-IP)**

Subcellular fractionation, followed by protein co-immunoprecipitation (co-IP) using the nuclear extract as input were performed as already described in ^1^. The antibodies employed for the co-IPs using LSD1 and GSE1 as baits were anti-LSD1 (Abcam, ab17721) and anti-GSE1 (Proteintech, 24947-1-AP), respectively.

**Differential Enrichment Analysis of Proteomics Data (DEP) of LSD1 interactome after LSD1 pharmacological inhibition**

The LSD1 interactome dataset upon 24 hours treatment with MC2580 was published in ^1^ and here it was re-analysed using DEP ^2^. For this analysis, the intensity in the heavy and light channels of the two SILAC replicates (Forward and Reverse) was used to calculate protein changes. A protein was considered significantly enriched/depleted if its log_2_ fold change (MC2580 vs DMSO) was greater than 1.5 or lower than -1.5 and its p-value (corrected for multiple testing) was < 0.05.

**Western blot analysis**

Cells were harvested, washed twice with PBS and re-suspended in RIPA buffer (10 mM Tris pH 8, 150 mM NaCl, 0.1% SDS, 1% Triton X-100, 1 mM EDTA, 0.1% Sodium Deoxicholate), supplemented with 1X EDTA-free Protease Inhibitors (Roche, 11836170001), 0.5 mM PMSF, 1 mM DTT and 1:50 Benzonase (250U/µl, E1014, Millipore). Re-suspended cells were incubated 45 minutes at room temperature on a rotating wheel and vortexed briefly at 15-minute intervals to facilitate cell lysis and protein extraction. Afterwards, samples were spun down at 16 060 x g for 30 minutes, at 4 °C in a bench-top centrifuge and supernatants -corresponding to the whole protein extracts- were collected and quantified by BCA assay (23225, Thermo Fisher Scientific). Around 30-40 µg of protein extract was mixed with Laemmli buffer containing 100 mM DTT and denatured for 5 minutes at 95 °C before loading on SDS-PAGE gel. Transfer of protein to PVDF or nitrocellulose membrane was performed at 100 V for 1 hour and 30 minutes at 4°C, or at 30 V overnight at 4 °C in Transfer buffer (Tris-Glycine) containing 10% methanol. Membranes were blocked in 10% BSA dissolved in TBS/0.1% Tween for 2 hours at room temperature. Primary antibodies were diluted in 5% BSA in TBS/0.1% Tween and incubated with the membrane overnight at 4 °C, or for 3 hours at room temperature. After three washes with TBS/0.1% Tween (5 minutes each), membranes were incubated with the corresponding secondary antibody in 5% BSA in TBS/0.1% Tween for 1 hour at room temperature. After three additional washes, protein signals were detected using the Enhanced Chemo Luminescence (ECL) method (Biorad, 1705061) and images were acquired with the ChemiDoc XRS+ System (Biorad, 1708265) or the Chemidoc^TM^ Touch Imaging System (Biorad). Quantitation of the bands was carried out with the ImageJ software. The following antibodies were used according to the company’s instruction at the indicated dilutions: anti-LSD1 (Abcam, ab17721, 1:1000), anti-GSE1 (Proteintech, 24947-1-AP, 1:600), anti-GFI1 (Santa Cruz Technology, sc-376949, 1:100), anti-H3 (Abcam, ab1791, 1:5000), anti-Vinculin (Sigma Aldrich, V9131, 1:5000), anti c-MYC (Santa Cruz Technology, sc-764, 1:200), anti-cleaved Caspase 3 (Cell Signalling Technology, #9661S, 1:1000), anti-total Caspase 3 (Cell Signalling Technology, #9662S, 1:1000), anti-V5 (Invitrogen, R960-25, 1:5000), anti α-Tubulin (Santa Cruz Technology, sc-32293, 1:500).

**RNA extraction and RT-qPCR**

Total RNA was extracted by using the RNeasy Mini kit (Qiagen, 74104) or the RNeasy Plus Mini kit (Qiagen, 74134) while reverse transcription was performed with SuperScript™ II Reverse Transcriptase (Invitrogen, 18064014) or the High-Capacity cDNA Reverse Transcription kit (Applied Biosystems, 4368814), according to the manufacturer’s protocols. Real time PCR was carried out in triplicate, in 20 μl final reaction volume constituted by 10 μl of 2X SYBR green Master Mix (Applied Biosystems, 4385614), 20 ng of cDNA retro-transcribed from the RNA, and 0.5 μM of each primer mix. Quantitative PCR amplifications were performed in the CFX96 Real-Time System (BioRad), using the following protocol: 1) 95 °C for 2 minutes, 2) 40 cycles at 95 °C for 10 seconds and 60 °C for 30 seconds. In *GSE1* KD experiments of BB104 *MLL-* translocated primary cells, real time PCR was performed in triplicate, in 10 μl final reaction volume composed of 5 μl of 2X SYBR green Master Mix, 25 ng of cDNA retro-transcribed from the RNA, and 0.5 μM of each primer mix. Quantitative PCR amplifications were carried out in the QuantStudio^TM^ 5 instrument (Applied Biosystems, A28140), using the following protocol: 1) 50 °C for 2 minutes, 2) 95 °C for 10 minutes, 3) 40 cycles at 95 °C for 15 seconds and 60 °C for 1 minute. The sequences of the primers are listed in the Table S7.

**Cell Transduction**

Lentiviral constructs were transiently transfected in HEK-293T cells by calcium phosphate transfection method ^3^ using the packaging plasmid pCMV-DR8.74 and the envelope plasmid pMD2G-VSVG. The retroviral vectors were, instead, transiently transfected with the same method in phoenix-AMPHO cells, together with the packaging plasmid pKAT. Supernatants from the HEK-293T or Phoenix-AMPHO cells were filtered with 0.45 µm filter and then ultra-centrifuged at 70 770 x g for 2 hours at 4 °C. The pellet -enriched of viral particles- was resuspended in culture medium and added to 2 x 10^6^ NB4 or THP-1 cells, previously plated in 6-well plates at 1 x 10^6^/ml concentration, together with 16 µg of polybrene. Cells were centrifuged at 1000 x g for 1 hour at room temperature in a swing-rotor centrifuge (Spin Infection) and then left incubating for 4 hours at 37 °C, prior to the addition of 2 ml growth medium. In the meantime, transfected HEK-293T or Phoenix-AMPHO cells were replaced with fresh medium that was, again, filtered and ultra-centrifuged for the second round of infection carried out the day after following the same protocol. Lentiviral and retroviral transductions were carried out in the Biosafety level 2 laboratory (BSL-2). In *GSE1* KD experiments of BB104 primary AML sample, cells were subjected to two rounds of infection: in each one cells were cultured overnight in viral supernatant supplemented with IL-3, G-CSF and TPO (all at a concentration of 20 ng/ml). Then, transduced cells were put under selection for 24 hours with 3 μg/ml puromycin, transferred to stromal layers (formed by MS5 cells) and cultured for additional 24h and 48h prior to analysis. For the following testss, leukaemia cells were isolated from stromal cells through disruption of the stromal layer by pipetting and then filtering the solution through a 75μm filter.

**Cell growth and viability assays**

For the growth curve, cultured cells were counted in Trypan Blue solution (T8154, Sigma Aldrich) using the TC20 automated cell counter from Bio-Rad (http://www.bio-rad.com/it-it/product/tc20-automated-cell-counter). The percentage of living cells was also assessed with this method.

**Cell cycle analysis by flow cytometry**

Infected cells were grown for the indicated time intervals and incubated in the presence of 33 µM of BrdU for 25 minutes, then harvested, washed once with PBS, and fixed with the addition of 70% ethanol dropwise while vortexing. Nuclei were denatured with 2N HCl for 20 minutes and then stained with a FITCH-conjugated anti-BrdU antibody (BD Biosciences, 347580) (5 μg/μl). Cells were washed with 1% BSA in PBS and re-suspended in Propidium Iodide (PI, 20 μg/ml) + RNase A (250 μg/ml). Stained cells were let on fluorescence activated cell sorting (FACS) tubes overnight at 4 °C before analyzing the samples with the FACS Celesta flow cytometer (BD Biosciences). Analysis of the cell cycle was performed using the function “cell cycle” of the FlowJo software.

**ChIP-qPCR**

For validation of specific genomic loci extrapolated from the ChIP-seq data, ChIP-qPCR was performed in NB4 and THP-1 cells as follows: 1 µl of DNA from ChIP and input samples was mixed with 10 µl of 2X SYBR green Master Mix (Applied Biosystems, 4385614) and 0.5 μM of each primer mix in a final reaction volume of 20 µl. Real time PCR was performed in three technical replicates and quantitative PCR amplifications were carried out in the CFX96 Real-Time System (BioRad), using the following conditions: 1) 95 °C for 2 minutes, 2) 40 cycles at 95 °C for 10 seconds and 60 °C for 30 seconds. The primers used are listed in the Table S7.

**Statistical analysis**

Most of the data are represented as mean + standard deviation (SD) and statistical analyses were carried out using two-tailed paired Student’s t-tests or one sample t-tests, unless others specified. The number (*n*) of biological replicates, the type of statistical analyses performed, and statistical significance are reported in the corresponding figures and/or figure legends for each experiment.

**Supplementary Figures**

**Fig. S1: MC2580 inhibitor decreases the protein expression of an exogenous form of GSE1.** A) Map of the pLEX_307 vector in which the insert of the *GSE1* coding sequence (NM_001134473.3) tagged with the V5 at the C-terminus, has been cloned. B) Map of the pLEX_306 vector in which the insert of *GSE1* coding sequence (NM_001134473.3) tagged with the V5 at the C-terminus, has been cloned. C) *Left panel:* Western Blot analysis of GSE1 and V5 in NB4 cells transduced with either the empty pLEX_307 (pLEX_307 EV), or the pLEX_307 *GSE1* and treated for 24 hours with MC2580 (2 μM) and DMSO. Vinculin was used as loading control. *Right panel:* Bar-graph displaying quantitation of the exogenous V5-tagged GSE1 protein level, normalized over the Vinculin, in 4 independent replicates of pLEX_307 EV and pLEX_307 *GSE1* transduced NB4 cells treated for 24 hours with either MC2580 (2 µM) or DMSO. The results are plotted as fold change of V5-GSE1 protein level in the treated sample normalised over the level of the corresponding protein in the control, DMSO-treated cells. The chart represents mean + standard deviation from n=4 biological replicates (one sample T-test, **p value<0.01). D) Western Blot analysis of GSE1 and V5 in pLEX_306 EV, pLEX_307 EV, pLEX_306 *GSE1* and pLEX_307 *GSE1* transduced in NB4 cells and treated for 24 hours with MC2580 (2 μM) and DMSO as negative control. Vinculin was used as loading control.

**Fig. S2: LSD1 inhibitors reduce GSE1 protein level in other AML subtypes beyond APL.** A) Western Blot analysis of GSE1 in THP-1 cells treated with MC2580 (2 µM), DDP-38003 (2 µM) and DMSO for 12 and 24 hours. Vinculin was used as loading control. M5 refers to the AML subtype to which these cells belong, according to the French-American-British (FAB) classification. B) Western Blot analysis of GSE1 and V5 in control (pLEX_307 EV) and *GSE1* over-expressing THP-1 cells generated by lentiviral transduction of the pLEX_307 *GSE1*. Cells were subsequently treated with MC2580 (2 μM) or DMSO for 24 hours. Vinculin was used as loading control. M5 refers to the AML subtype in which these cells are included, according to the FAB classification. C) Western Blot analysis of GSE1 in SKNO-1 cells treated with MC2580 (2 µM), DDP-38003 (2 µM) and DMSO for 12 and 24 hours. Vinculin was used as loading control. M2 refers to the AML subtype in which these cells are included, according to the FAB classification. D) Western Blot analysis of GSE1 in OCI-AML2 cells treated with MC2580 (2 µM), DDP-38003 (2 µM) and DMSO for 12 and 24 hours. Vinculin was used as loading control. M4 refers to the AML subtype to which these cells belong, according to the FAB classification. E) Western Blot analysis of GSE1 in BB104 *MLL-* translocated primary patient cells ^4^ treated with MC2580 (2 µM), DDP-38003 (2 µM) and DMSO for 24 hours. Vinculin was used as loading control.

**Fig. S3: Analysis of GSE1 protein stability in NB4 cells.** *Left panel:* Time-resolved Western Blot analysis of GSE1 and c-MYC protein levels in NB4 cells at 3-, 6-, 9- and 12-hours post-treatment with cycloheximide (CHX) (0.1 mg/ml) and Ethanol (EtOH) as negative control. Vinculin was used as loading control. *Right panel:* Line-plot displaying the percentage of GSE1 protein level normalized over Vinculin in cycloheximide-treated NB4 cells, compared to control, EtOH-treated ones.

**Fig. S4: Assessment of the efficiency of MC2580 treatment in the absence of cycloheximide as control for the results displayed in Figure 1H.** A) Time-resolved Western Blot analysis of GSE1 and c-MYC levels in NB4 cells treated with EtOH (negative control for CHX), in combination with either MC2580 (2 µM) or DMSO for 3, 6, 9 and 12 hours as control of the first biological replicate of the experiment displayed in Figure 1H. Vinculin was used as loading control. B) Western Blot analysis of GSE1 and c-MYC levels in NB4 cells treated with EtOH in combination with either MC2580 (2 µM) or DMSO for 3, 6, 9 and 12 hours as control of the second biological replicate of the experiment in Figure 1H. Vinculin was used as loading control.

**Fig. S5: *GSE1* knock-down (KD) increases the percentage of cells in G1.** A) Representative cell cycle flow cytometry analysis of NB4 cells at 48- and 72-hours post-transduction with either an empty pLKO.1 puro vector (EV) or the pLKO.1 puro vector, in which the shA1 and shB2 constructs targeting *GSE1* are cloned. B) Percentage of shA1, shB2 and EV-transduced cells in each cell cycle phase at 48 and 72 hours upon transduction. The chart represents mean + standard deviation (SD) of two biological replicates (n=2).

**Fig. S6: Supplementary data for the *in vivo* experiment to assess the effect of *GSE1* KD in the tumour growth formation of NB4 cells in NOD/SCID gamma (NSG) mice.** A) Percentage of viable NB4 cells 24 hours after transduction with shA1 and shB2 targeting *GSE1* or with the empty pLKO.1 puro vector (EV), used as negative control**.** B) Western blot (WB) analysis to assess GSE1 protein level in NB4 cells used for transplantation in NOD/SCID gamma (NSG) mice. WB analysis was performed 48 hours post- transduction with either EV or the same vector containing the shA1 and shB2 constructs. Vinculin was used as loading control. C) Complete tumour growth curve from fig. 2F extended until day 47 in NSG mice transplanted subcutaneously with EV- and *GSE1* KD- transduced NB4 cells. Graph represents mean ± SD of the tumour volume in each condition (n=8, 8 distinct mice for each group). D) Western Blot analysis *(left panel)* and associated quantification *(right panel)* of GSE1 protein in tumours extracted from NSG mice injected subcutaneously with NB4 cells transduced with *GSE1* KD (shA1 and shB2) or EV as negative control. Tumour samples were collected when mice were sacrificed: tumour developed by EV-transduced cells was collected at day22, shB2 #7 at day29, shA1 #2 and shB2 #3 at day32, shA1 #6 and shB2 #5 at day36, shA1 #4 and shB2 #4 at day39, shA1 #7 at day43.

**Fig. S7: Reactome analysis of the 422 up-regulated genes upon *GSE1* KD.** Bar-graph displaying the Reactome results on the enriched pathways associated with the 422 up-regulated genes upon *GSE1* depletion in NB4 cells. Reactome analysis and calculation of the statistically significant pathway terms was performed with EnrichR ^5^ (Adjusted p-value < 0.05). The x-axis displays the -log_10_ of the adjusted p-value for each significant term. The light blue dashed line indicates the significant threshold used to define the enriched pathways.

**Fig. S8: Analysis of differentially expressed genes upon MC2580 treatment in NB4 cells.** Volcano-plot displaying the up- and down- regulated genes 24 hours post- MC2580 treatment. On the x- axis, the log_2_ fold change (FC) values of LSD1-inhibited cells versus DMSO-treated ones are displayed, while the y-axis displays the -log_10_ of the adjusted p-value (padj) for each gene. FC value was calculated with DEseq2 program ^6^, using two biological replicates for each condition (n=2).

**Fig. S9: RT-qPCR analysis validates RNA-seq results.** A) RT-qPCR analysis on a panel of genes in NB4 cells treated for 24 hours with MC2580 (2 µM), DDP-38003 (2 µM) and DMSO as control. Ct values of different genes are normalized against *GAPDH*. The results are plotted as fold change (FC) of the mRNA levels in the treated conditions compared to DMSO. Chart represents mean + standard deviation (SD) (n=3 biological replicates, one sample T-test, ***p-value<0.001, **p-value<0.01, *p-value<0.05). B) RT-qPCR analysis on a panel of genes at 48 hours post- transduction with shA1, shB2 and the empty vector pLKO.1 puro (EV) as negative control in NB4 cells. Ct values of the different genes are normalized over *GAPDH*. The results are plotted as FC of the mRNA levels in the *GSE1* KD samples over the corresponding levels in the EV-transduced cells. Bar-graph represents mean + SD (n=3 biological replicates, one sample T-test, ***p-value<0.001, **p-value<0.01, *p-value<0.05).

**Fig. S10: Effect of MC2580 treatment on H3K4me1, H3K4me2 and H3K27ac localisation at the GSE1-bound genomic loci.** Heatmap representation of the normalized ChIP-seq intensities of H3K4me1, H3K4me2 and H3K27ac at ±5 kb around the center of the GSE1-bound loci, 24 hours after treatment with MC2580 (2 μM) and DMSO. The heatmap is divided in different groups, as classified and reported in Figure 4D. The H3K4me1, H3K4me2 and H3K27ac ChIP-seq data used has already been published in ^1^.

**Fig. S11: Analysis of genomic categories associated to the regions where GSE1 binding is reduced or abolished upon LSD1 inhibition shows higher percentage of promoter regions.** Genome annotation of the V5-GSE1 ChIP-seq peaks in the different genomic categories described in Figure 4D.

**Fig. S12: Ingenuity Pathway Analysis (IPA) of “Physiological System Development and Function” terms upon LSD1 inhibition and *GSE1* KD.** Representation of the top five “Physiological System Development and Function” IPA terms extracted from the analysis of the differentially expressed genes (DEGs) upon MC2580 treatment and *GSE1* KD.

**Fig. S13: Effects of *GSE1* knock-down (KD) on viability and apoptosis of THP-1 cells.** A) Bar-graph displaying the percentage of dead cells in THP-1 transduced with shA1 and shB2 targeting *GSE1* or with the empty vector pLKO.1 puro (EV) as negative control. The analysis was performed at 24-hour time intervals, starting from 24 until 168 hours following infection. Cell viability was measured by Trypan blue staining. Chart represents mean + SD (n=2 biological replicates). B) Western blot analysis of GSE1, cleaved and total caspase-3 in cells transduced for 72 hours with either the EV or shA1 and shB2. Tubulin was used as loading control.

**Fig. S14: *GSE1* over-expression (OE) in NB4 cells increases c-KIT stem/progenitor marker.** A) Flow cytometry analysis to estimate the percentage of c-KIT positive cells in control (EV) and *GSE1*-overexpressing (OE) NB4 cells, treated for 24 hours with MC2580 (2 μM). *GSE1* OE NB4 cells were generated by transducing the pLEX_307 *GSE1*, while control cells were transduced with the empty vector (EV). Chart represents mean + standard deviation (SD) (n=4 biological replicates; paired T-test, ***p-value<0.001). B) *Left panel:* The dot-plot displays the percentage of c-KIT positive cells in EV and *GSE1* OE NB4 cells from one of the three biological replicates and used as representative result. *Right panel:* Bar-graph of the quantitation of the percentage of c-KIT positive cells in EV and *GSE1* OE NB4 cells. Chart represents mean + standard deviation (SD) (n=3 biological replicates; unpaired T-test, ****p-value<0.0001).

**Fig. S15: *GSE1* OE does not rescue the down-regulation of c-KIT triggered by MC2580 in THP-1 cell line.** Flow cytometry analysis of c-KIT positive cells in control (EV) and *GSE1* OE THP-1 cells treated for 24 hours with MC2580 (2 μM). *GSE1* OE THP-1 cells were produced by lentiviral transduction of the pLEX_307 *GSE1*, while control cells were transduced with an empty vector (EV). The chart represents mean + standard deviation (SD) (n=3 biological replicates).

**Fig. S16: Biological processes (BP) of genes with expression levels negatively correlating with *GSE1* and displaying r < -0.5.** GO analysis of the enriched biological process (BP) terms on the list of genes whose expression levels are significantly negatively correlated (FDR<0.05) with those of *GSE1* in the TCGA ^7^ (*left panel*) and Tyner *et al.* ^8^ datasets (*right panel*) and display r < -0.5. Bar graphs represent the top 10 enriched BP terms ranked according to the Enriched Ratio. Y-axis lists the significant BP terms while x- axis the Enrichment Ratio.

**Fig. S17: Survival curves of AML patient samples with different *GSE1* expression levels.** A) Kaplan-Meier survival curves of the cohorts of AML patients from TCGA ^7^ (*left panel*) and Tyner *et al.* ^8^ (*right panel*) datasets. Patients were grouped according to the mRNA levels of *GSE1* (i.e., *GSE1* above median value: red line; *GSE1* below median value: blue line). B) Kaplan-Meier survival curves of the subset of patients with M3-AML pooled from the two datasets and grouped according to *GSE1* mRNA levels (i.e., *GSE1* above median value: black line; *GSE1* below median value: red line).

**Supplementary Table Legends**

**Table S1:** Gene expression profiling by RNA-seq in NB4 cells transduced for 48 hours with shA1 and shB2 targeting *GSE1* or the empty pLKO.1 lentiviral vector as negative control.

**Table S2:** Gene Ontology (GO) analysis of the Biological Processes (BP) enriched after *GSE1* knock-down (KD). *Spreadsheet 1*: Complete list of BP terms obtained from the GO analysis of the up-regulated genes after *GSE1* KD. Statistically significant terms are displayed in green (adjusted p-value < 0.05). GO analysis was performed through EnrichR ^5^. *Spreadsheet 2*: List of BP terms extrapolated from the GO analysis of the down-regulated genes after *GSE1* KD. GO analysis was performed through EnrichR ^5^.

**Table S3:** RNA-seq analysis of NB4 cells treated for 24 hours with 2 μM of MC2580 or DMSO.

**Table S4:** Ingenuity Pathway Analysis (IPA) of the differentially expressed genes (DEG) after LSD1 pharmacological inhibition and *GSE1* knockdown (KD). *Spreadsheet 1*: List of the IPA pathways extrapolated from the analysis of the DEG after 24h treatment with MC2580 (2 μM). Statistically significant IPA pathways are displayed in green (-log_10_ p-value > 2). Associated biological processes for each IPA term are annotated manually and indicated only for the significant IPA pathways. *Spreadsheet 2*: List of the IPA pathways obtained from the analysis of the DEG upon *GSE1* KD. Statistically significant IPA pathways are displayed in green (-log_10_ p-value > 2). Associated biological processes for each IPA term are annotated manually and indicated only for the significant IPA pathways.

**Table S5:** Annotation of the V5-GSE1 binding sites in the genomic categories “Regions bound by GSE1 similarly in both DMSO- and MC2580- treated cells” (*Spreadsheet 1*), “Regions bound by GSE1 only in DMSO condition” (*Spreadsheet 2*), “GSE1-bound regions only upon MC2580” (*Spreadsheet 3*) and “GSE1-bound regions that display reduced binding upon MC2580” (*Spreadsheet 4*).

**Table S6:** List of genes whose expression levels negatively correlates with *GSE1* in the Tyner *et al.* dataset ^8^ (*Spreadsheet 1*) and TCGA ^7^ (*Spreadsheet 2*).

**Table S7:** List of primers used for the RT-qPCR (*Spreadsheet 1*) and the ChIP-qPCR (*Spreadsheet 2*) analyses.

**References**

1	Ravasio R, Ceccacci E, Nicosia L, Hosseini A, Rossi PL, Barozzi I *et al.* Targeting the scaffolding role of LSD1 (KDM1A) poises acute myeloid leukemia cells for retinoic acid-induced differentiation. *Sci Adv* 2020. doi:10.1126/sciadv.aax2746.

2	Zhang X, Smits AH, Van Tilburg GBA, Ovaa H, Huber W, Vermeulen M. Proteome-wide identification of ubiquitin interactions using UbIA-MS. *Nat Protoc* 2018. doi:10.1038/nprot.2017.147.

3	Kingston RE, Chen CA, Rose JK. Calcium Phosphate Transfection. *Curr Protoc Mol Biol* 2003. doi:10.1002/0471142727.mb0901s63.

4	Maiques-Diaz A, Spencer GJ, Lynch JT, Ciceri F, Williams EL, Amaral FMR *et al.* Enhancer Activation by Pharmacologic Displacement of LSD1 from GFI1 Induces Differentiation in Acute Myeloid Leukemia. *Cell Rep* 2018. doi:10.1016/j.celrep.2018.03.012.

5	Chen EY, Tan CM, Kou Y, Duan Q, Wang Z, Meirelles G V. *et al.* Enrichr: Interactive and collaborative HTML5 gene list enrichment analysis tool. *BMC Bioinformatics* 2013. doi:10.1186/1471-2105-14-128.

6	Love MI, Huber W, Anders S. Moderated estimation of fold change and dispersion for RNA-seq data with DESeq2. *Genome Biol* 2014. doi:10.1186/s13059-014-0550-8.

7	Cancer Genome Atlas Research Network, Ley TJ, Miller C, Ding L, Raphael BJ, Mungall AJ *et al.* Genomic and Epigenomic Landscapes of Adult De Novo Acute Myeloid Leukemia. *N Engl J Med* 2013; **368**: 2059–2074.

8	Tyner JW, Tognon CE, Bottomly D, Wilmot B, Kurtz SE, Savage SL *et al.* Functional genomic landscape of acute myeloid leukaemia. *Nature* 2018; **562**: 526–531.
